# Global photosynthetic capacity jointly determined by enzyme kinetics and eco-evo-environmental drivers

**DOI:** 10.1016/j.fmre.2023.12.011

**Published:** 2024-02-06

**Authors:** Zhengbing Yan, Matteo Detto, Zhengfei Guo, Nicholas G. Smith, Han Wang, Loren P. Albert, Xiangtao Xu, Ziyu Lin, Shuwen Liu, Yingyi Zhao, Shuli Chen, Timothy C. Bonebrake, Jin Wu

**Affiliations:** a State Key Laboratory of Vegetation and Environmental Change, Institute of Botany, Chinese Academy of Sciences, Beijing 100093, China; b School of Biological Sciences, The University of Hong Kong, Hong Kong 999077, China; c China National Botanical Garden, Beijing 100093, China; d Department of Ecology and Evolutionary Biology, Princeton University, Princeton NJ 08544, USA; e Department of Biological Sciences, Texas Tech University, Lubbock TX 79409, USA; f Ministry of Education Key Laboratory for Earth System Modelling, Department of Earth System Science, Tsinghua University, Beijing 100084, China; g Joint Centre for Global Change Studies, Tsinghua University, Beijing 100084, China; h Department of Biology, West Virginia University, Morgantown WV 26506, USA; i Department of Ecology and Evolutionary Biology, Cornell University, Ithaca NY 14853, USA; j Department of Ecology and Evolutionary Biology, University of Arizona, Tucson AZ 85721, USA; k Institute for Climate and Carbon Neutrality, The University of Hong Kong, Hong Kong 999077, China; l State Key Laboratory of Agrobiotechnology, The Chinese University of Hong Kong, Shatin, Hong Kong 999077, China

**Keywords:** Global carbon cycling, Leaf photosynthetic capacity, Enzyme kinetics, Eco-evolutionary optimality, Ecophysiology, Climate, Leaf traits, Belowground resource constraint

## Abstract

Accurate understanding of global photosynthetic capacity (i.e. maximum RuBisCO carboxylation rate, *V*_c, max_) variability is critical for improved simulations of terrestrial ecosystem photosynthesis metabolisms and carbon cycles with climate change, but a holistic understanding and assessment remains lacking. Here we hypothesized that *V*_c, max_ was dictated by both factors of temperature-associated enzyme kinetics (capturing instantaneous ecophysiological responses) and the amount of activated RuBisCO (indexed by *V*_c, max_ standardized at 25 ℃, *V*_c, max25_), and compiled a comprehensive global dataset (*n* = 7339 observations from 428 sites) for hypothesis testing. The photosynthesis data were derived from leaf gas exchange measurements using portable gas exchange systems. We found that a semi-empirical statistical model considering both factors explained 78% of global *V*_c, max_ variability, followed by 55% explained by enzyme kinetics alone. This statistical model outperformed the current theoretical optimality model for predicting global *V*_c, max_ variability (67%), primarily due to its poor characterization on global *V*_c, max25_ variability (3%). Further, we demonstrated that, in addition to climatic variables, belowground resource constraint on photosynthetic machinery built-up that directly structures the biogeography of *V*_c, max25_ was a key missing mechanism for improving the theoretical modelling of global *V*_c, max_ variability. These findings improve the mechanistic understanding of global *V*_c, max_ variability and provide an important basis to benchmark process-based models of terrestrial photosynthesis and carbon cycling under climate change.

## Graphical abstract

**Figure:**
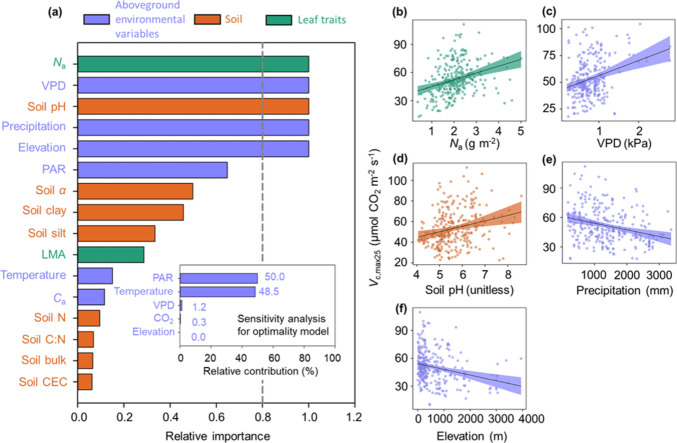

## 1 Introduction

Terrestrial photosynthesis is the largest carbon flux exchange between biosphere and atmosphere and exerts an important role in buffering atmospheric CO_2_ growth [1,2]. Under many real world conditions, photosynthesis is expected to be limited by the maximum carboxylation rate of RuBisCO in the chloroplasts (*V*_c, max_) [3], [4], [5]. *V*_c, max_ shows a large variability globally and is affected by multiple environmental and biotic variables, such as climate, edaphic properties and leaf traits [6], [7], [8], [9], all of which will be altered by future climate and land use changes. Although predicting *V*_c, max_ variability has received much scientific attention [8,[10], [11], [12], a holistic understanding of what drives global *V*_c, max_ variability remains lacking.

Traditionally, empirically derived trait coordinated relationships and theory-based optimality models are used to explain *V*_c, max_ variability [3,[8], [9], [10]. The trait-based approach suggests that *V*_c, max_ can be estimated using its empirical relationships with other leaf biochemical traits (e.g., leaf N content per unit leaf area (*N*_a_) and leaf chlorophyll content) that tightly correlate with photosynthetic biochemistry [3,^7^,10]. However, mounting evidence suggests that trait-*V*_c, max_ relationships vary with plant growing environments, seasons and other biotic variables [13], [14], [15], [16], which further induces large uncertainties in representing *V*_c, max_ in terrestrial biosphere models (TBMs). Additionally, the trait-based approach is ultimately based on empirical relationships, lacking mechanistic foundation that is critical for long-term predictions under global change.

Recently, theoretical optimality models have been increasingly used for explaining and modelling large-scale *V*_c, max_ variability [8,^17^,18]. The models were built based on eco-evolutionary first-principles [13,[19], [20], [21], hypothesizing that plants can adjust their photosynthetic biochemistry for maximizing their net carbon gain, which is the difference between photosynthetic carbon gain and all carbon costs of building and maintaining the photosynthetic machinery [13,22]. Because optimality models are mechanistic and prognostic, they have been suggested as a novel means to infer *V*_c, max_ variability [18,23]. To support its validity is a recent theoretical study [8], demonstrating that optimality models driven by aboveground environmental variables (i.e., temperature, incoming photosynthetically active radiation (PAR), vapor pressure deficit (VPD), atmospheric CO_2_ concentration (*C*_a_), and elevation (a proxy of atmospheric pressure)) alone can accurately capture global *V*_c, max_ variability. However, it remains contentious whether the optimality models could accurately predict *V*_c, max_ at a standardized temperature (e.g. *V*_c, max25_, *V*_c, max_ standardized at 25 ℃) [8,12]. Some studies suggested that the optimality model could be more appropriate to predict *V*_c, max_ during the growing-season temperature (*V*_c, maxTg_) rather than the *V*_c, max25_, because growing-season temperature is the temperature regularly experienced by plants rather than a standard temperature, which may be atypical for that environment [8,24]. In contrast, some other studies hold the opposite viewpoint that the optimality model could also accurately predict *V*_c, max25_, but with the use of limited dataset and the growing-season temperature as the proxy of leaf measurement temperature when calculating *V*_c, max25_ from the measured *V*_c, max_ [12,25]. Therefore, despite its promising application in plant ecophysiology and modelling studies, a more rigorous and holistic assessment remains needed. Meanwhile, several empirical studies demonstrate the critical role of belowground resource availability on regulating large-scale *V*_c, max_ variability [26,27]. For example, a large fraction of leaf nitrogen is allocated to RuBisCO enzyme, which is ultimately related to soil nutrient availability and associated plant evolved strategies for root nitrogen uptake [13,27]. With these, it remains unclear whether and how belowground environmental conditions exert a role in constraining photosynthetic machinery built-up in the theoretical optimality modelling framework.

On the other hand, *V*_c, max_ is the product of catalytic rate and amount of RuBisCO enzyme (indicated by *V*_c, max25_) in the chloroplasts [17,28]. This can be interpreted as two key factors governing *V*_c, max_ variability, namely the temperature-associated enzyme kinetics of a given *V*_c, max25_ and the eco-evo-environmental drivers of *V*_c, max25_. The second category, the eco-evo-environmental drivers of *V*_c, max25_, stands in contrast with pure enzyme kinetics. Within this second category, *V*_c, max25_ variability is associated with environmental factors and leaf traits [3,^6^,^10^,29], which can influence plant photosynthetic processes, the construction and maintenance of photosynthetic apparatus, carbon uptake capacity and then the subsequent responses to changing ambient conditions [30,31]. Thus, incorporating the relationships between *V*_c, max25_ variability and these environmental factors and leaf traits could further enhance the characterization of global *V*_c, max_ variability.

The goal of this study is to improve the process understanding of global *V*_c, max_ variability by first assessing the relative role of enzyme kinetics and eco-evo-environmental drivers of *V*_c, max25_, and then exploring the way to improve current optimality models for characterizing global *V*_c, max_ variability. Specifically, we test the hypothesis that global patterns of *V*_c, max_ are driven by (1) enzyme kinetics, (2) eco-evo-environmental drivers of *V*_c, max25_, or (3) both enzyme kinetics and the eco-evo-environmental drivers of *V*_c, max25_. Additionally, we test whether current optimality model explains global pattern of *V*_c, max_, once *V*_c, max_ has been standardized to 25 ℃ (to remove the temperature response of *V*_c, max_). Last, we test whether including belowground resource constraints through edaphic properties on photosynthetic machinery would improve current optimality modelling of global variability in both *V*_c, max_ and *V*_c, max25_. To test these hypotheses, we collated a comprehensive global dataset of *V*_c, max_ for C_3_ plants with concurrent measurements of environmental variables and key leaf traits, and then integrated this unique global dataset with both statistical and optimality modelling analyses.

## 2 Materials and methods

### 2.1 Field dataset of *V*_c, max_, climate and edaphic variables, and leaf traits

To compile the global *V*_c, max_ dataset of C_3_ plants (Fig. S1), we turned to the following three data sources: two global data sources assembled by Smith et al. [8] and Peng et al. [12], respectively, and one data source from three contrasting forest ecosystems in China [16]. The two global data sources were mainly derived from earlier compilations by Meir et al. [32], Domingues et al. [33,34], Cernusak et al. [35], Walker et al. [3], Atkin et al. [36], Maire et al. [37], Bahar et al. [26], Smith & Dukes [38], Dong et al. [39], Wang et al. [40], Bloomfield et al. [41], Xu et al. [42] and the TRY plant trait database. It is worthy to note, when compiling this dataset, we only retained the records with concurrent measurement of leaf temperature. This also represents one major difference between our compiled data and those compiled by Peng et al. [12], in which Peng et al. [12] used the growing-season temperature as the proxy of leaf measurement temperature for those *V*_c, max_ records lack of the information on leaf measurement temperature (details about the cross-comparison between ours and Peng et al. [12] are shown in Fig. S2 and in discussion Section 2). In our data, all *V*_c, max_ records came from natural vegetation, including 7339 measurements from 2250 species and 428 sites covering all major biomes worldwide. In order to quantify the separate and joint effects of enzyme kinetics and eco-evo-environmental drivers of *V*_c, max25_ on global *V*_c, max_ variability, only those *V*_c, max_ records with concurrent measurements of environmental variables and leaf traits (i.e., LMA and *N*_a_) were chosen. Totally, 5748 measurements from 281 sites met these selection criteria.

Besides, we extracted six climate-related variables (i.e. temperature, VPD, PAR, *C*_a_, elevation, and precipitation). For each climate variable, the average value across the full growing season (defined as all months with mean monthly air temperature higher than 0 ℃) was calculated for each site [8]. These include (1) the mean growing-season temperature (*T*_g_) and precipitation extracted using the corresponding latitude and longitude from monthly, 0.5 degree resolution data of 1901-2015 provided by Climatic Research Unit [43]; (2) VPD and PAR calculated from the CRU data using SPLASH model; (3) *C*_a_ derived primarily from original records in earlier compilations, but when there was no *C*_a_ record, it was estimated using corresponding value from global average estimates by NASA GISS model (https://data.giss.nasa.gov/modelforce/ghgases/); and (4) elevation was partly derived from original records, but when there was no record, it was extracted from 0.5 degree resolution data from WFDEI meteorological forcing dataset [44]. Notably, *T*_g_ and precipitation were three-dimensionally interpolated to the actual site locations using Geographically Weighted Regression (GWR) following Peng et al. [12], while VPD and PAR were adjusted to the actual elevation following Smith et al. [8].

Moreover, we extracted ten edaphic variables, including pH, carbon (C) content, nitrogen (N) content, C:N ratio, Priestley-Taylor coefficient (*α*; this variable indicated plant-available surface moisture, and was calculated as the ratio of actual evapotranspiration to equilibrium evapotranspiration. Equilibrium evapotranspiration refers to theoretical value of evaporation from a wet surface to a saturated atmosphere), cation exchange capacity (CEC), silt content, clay content, sand content, and bulk density. *α* of each 0.5 degree resolution was calculated using the SPLASH model run at a monthly timescale [45]. Other edaphic variables, comprehensively reflecting soil physical and chemical conditions, were extracted from a 250-m resolution global data at the top 30 cm depth provided by ISRIC SoilGrids database (www.soilgrids.org) based on site-specific latitude and longitude.

### 2.2 Field dataset of *V*_c, maxTmeas_ and *V*_c, max25_

With *V*_c, max_ derived at its measurement temperature (*T*_meas_, ℃), or *V*_c, maxTmeas_, we calculated *V*_c, max_ respectively at *T*_g_ ($V_{c,\;\text{maxTg}}$, ℃) and 25 ℃ (*V*_c, max25_), using a modified Arrhenius function (see Eqs. (1)-(2)) that described the instantaneous response of enzyme kinetics to any given temperature [28]. Given a reference temperature *T*_0_ (℃), *V*_c, max_ at temperature *T*_1_ (℃) can be computed as

(1) $$V_{c,\;\text{max}T_{1}}=V_{c,\;\text{max}T_{0}}\times f\left(T_{0},T_{1}\right)$$

where

(2) $$f\left(T_{0},T_{1}\right)=e^{\frac{H_{a}\left(T_{1}-T_{0}\right)}{R\left(T_{0}+273.15\right)\left(T_{1}+273.15\right)}}\times \frac{1+e^{\frac{\left(T_{0}+273.15\right)\left(\Delta S\right)-H_{d}}{R\left(T_{0}+273.15\right)}}}{1+e^{\frac{\left(T_{1}+273.15\right)\left(\Delta S\right)-H_{d}}{R\left(T_{1}+273.15\right)}}}$$

where *H*_a_ is the activation energy (71,513 J mol^−1^), *R* is the universal gas constant (8.314 J mol^–1^ K^–1^), *H*_d_ is the deactivation energy (200,000 J mol^−1^), and *∆S* is an entropy term (J mol^–1^ K^–1^) calculated following Kattge and Knorr (2007) [28].

(3) $$\Delta S=-1.07\times T_{g}+668.39$$

Spurious correlations may arise because the same temperature response function was applied to both observed and modeled *V*_c, max_. Smith et al. [8] has examined this issue and found that the temperature scalar did yield some but low spurious correlations. We also compared models against *V*_c, maxTmeas_ that did not subject to the temperature scalar issue, and found similar results as those for *V*_c, maxTg_ (Fig. S3), suggesting that spurious correlation effects induced by the temperature scalar function would not affect our result interpretations.

### 2.3 Statistical and optimality modelling approaches for predicting global *V*_c, maxTg_ variability

To improve the process understanding of global *V*_c, max_ variability, we cross-compared a statistical approach with an optimality modelling approach for predicting *V*_c, maxTg_ and *V*_c, max25_, respectively. The statistical model examined the separate and joint effects of the two factors in explaining *V*_c, maxTg_ variability, with two levels of analyses. In the first level, we used the temperature-associated enzyme kinetics response assuming no intra- or inter-specific variability in *V*_c, max25_ across global C_3_ plants. In the second level, we added temperature-associated enzyme kinetics to site-specific *V*_c, max25_ derived from its empirical relationship with both environmental variables and leaf traits, assuming that large geographical variability in *V*_c, max25_ was correlated with their living environments [6,29] and leaf traits [3,10]. The optimality modelling relies on an established optimality theory [8], and is able to infer *V*_c, max,Tg_ from five aboveground environmental variables (i.e., temperature, PAR, VPD, elevation and *C*_a_). The current optimality model by default assumes that the cost of building photosynthetic machinery is independent of species and the belowground environmental condition. To account for variable costs, particularly the cost of nutrient acquisition [27,37], we built and evaluated a modified optimality model that considered the belowground resource constraints through edaphic variables on the unit cost of building and maintaining the photosynthetic machinery.

All analyses were performed at site-mean level by averaging all measurements of each site. Details of these modelling analyses are shown below. For simplicity, we primarily focused on the data analyses for the subset dataset with concurrent measurements of environmental variables and leaf traits in the main text, while the results from the data analyses for the entire dataset (solely including the aboveground environmental measurements) are shown in the supplementary materials with similar findings (Figs. S4-S6).

#### 2.3.1 Statistical modelling approach for predicting global V_c, maxTg_ variability

There are two levels of statistical modelling analyses:

(1) Quantifying the effect of enzyme kinetics on global *V*_c, maxTg_ variability. To assess the effect of enzyme kinetics alone on explaining global *V*_c, maxTg_ variability, we first derived the site-mean *V*_c, max25_ based on field measurements, and then calculated the average *V*_c, max25_ across all sites, assuming that all the C_3_ plants shared the same *V*_c, max25_ as this average value. Afterwards, for each site, we calculated the modelled $V_{c,\;\text{maxTg}}$ using Eqs. 1-3 that multiplied this global-average *V*_c, max25_ with $f(25,\;T_{g})$.

(2) Quantifying the joint effects of enzyme kinetics and eco-evo-environmental drivers of *V*_c, max25_ on global *V*_c, maxTg_ variability. We first built a multiple linear regression model of *V*_c, max25_ with site-specific environmental variables and leaf traits as the input (Table S1), and then added this modelled *V*_c, max25_ with temperature-associated enzyme kinetics (Eqs. 1-3) for deriving *V*_c, maxTg_ of each site. The coefficients of predictors in the multiple linear regression were determined using the Ordinary Least-Squares method by minimizing the root mean square error (RMSE) between the observed and predicted *V*_c, max25_ values. Specifically, *V*_c, max25_ and *V*_c, maxTg_ modelling were built and evaluated using a repeated cross-validation method, including four steps: (1) the full dataset was split into calibration and independent validation subsets using a 5-fold cross-validation with 100 repetitions; (2) for each repetition, the calibration subset was used to build the model, with the validation subset for model evaluation; (3) the modelled *V*_c, max25_ for each validation subset was calculated and averaged across all 100 repetitions to obtain the ensemble predicted value for each record; and (4) the ensemble predicted *V*_c, max25_ and their associated $f(25,\;T_{g})$, were used together to estimate *V*_c, maxTg_ of each record.

#### 2.3.2 The optimality modelling approach for V_c, maxTg_

(1) *The optimality theory*

The optimality model shown in Smith et al. [8] is based on two separate optimization processes. The first process involves the leaf photosynthetic process, and the total carbon gain is derived from leaf assimilation rate (*A*), whereas the respiration carbon cost is required to build and maintain the photosynthetic machinery, including the light harvesting component (via *J*_max_) and the carbon reduction component (via *V*_c, max_) [13]. Thus, the first optimization is expressed as maximizing the net carbon gain, which is the difference between *A* and the carbon costs associated with maintaining *V*_c, max_ and *J*_max_ [8]. The second optimization process applies the least-cost theory to find the optimal leaf internal to external CO_2_ ratio (*χ*) at the lowest carbon cost to maintain photosynthetic machinery (*V*_c, max_) and water transpiration (*E*) at a given assimilation rate (*A*) [13]. These two optimality conditions can be expressed as:

(4) $$\max_{V_{c,\;\max},\;J_{\max}}\left(A-b\times V_{c,\;\text{max}}-d\times J_{\text{max}}\right)$$

(5) $$\min_{\chi }\left(a\times \frac{E}{A}+b\times \frac{V_{c,\;\text{max}}}{A}\right)$$

where *a, b*, and *d* are the dimensionless carbon cost factors for *E, V*_c, max_ and the maximum electron transport rate (*J*_max_), respectively. Based on an assumption that *V*_c, max_ is linearly proportional to *J*_max_ (i.e. *V*_c, max_ = *e*×*J*_max,_ where *e* is the ratio of *V*_c, max_ to *J*_max_), the total carbon costs for both *V*_c, max_ and *J*_max_ could then be expressed by the carbon cost for *J*_max_ only. Thus, the first optimality condition can further be reduced to

(6) $$\max_{J_{\max}}\left(A-c\times J_{\text{max}}\right)$$

where *c* is defined as the total unit carbon cost of building and maintaining the two components of photosynthetic machinery. By building upon these two independent optimizations, *V*_c, maxTg_ can further be inferred with solely environmental inputs from *T*_g_, PAR, VPD, *C*_a_, and elevation. The key mathematical formulations have been introduced in Smith et al. (2019) [8]. Here we provided the details about the optimality model in the supporting method S1.

(2) Evaluating the optimality model with a constant or dynamic cost

The marginal cost *c* (i.e., $\frac{\partial A_{j}}{\partial J_{max}}$) reflects the fundamental tradeoff between photosynthetic carbon gain (i.e., $A_{j}$) and the relevant cost for photosynthetic machinery built-up and maintenance (i.e., *J*_max_ and *V*_c, max_, which in this study are assumed to be linearly related), and is an important parameter for predicting *V*_c, max_ in the optimality model (Eqns 6 and S10). In the optimality model of Smith et al., [8], *c* was parameterized with a default globally constant at 0.053. However, as several empirical and theoretical studies demonstrated, the costs in constructing and maintaining the photosynthetic machinery could also depend on environmental factors and leaf traits [13,^22^,^26^,^27^,46]. To explore whether incorporating the constraints from environmental variables and leaf traits on the cost factor *c* can improve the current optimality model performance, we parameterized *c* under four scenarios (Table S2 and S3): (1) a globally constant *c* at 0.053 across all sites as used in the current optimality model [8], and a site-specific variable *c* respectively constrained by (2) the edaphic variables alone, (3) both environmental (i.e. climatic and edaphic) variables and leaf traits, and (4) the five most important variables (i.e. *N*_a_, VPD, soil pH, precipitation and elevation) identified from the statistical modelling (Fig. 4). To differentiate these four optimality models, we called (1) as optimality-constant (with a fixed *c*) and (2-4) as optimality-dynamic models (with a variable *c*).

For the three optimality-dynamic models, the cost factor *c* was fitted with a four-step approach illustrated below. First, the full dataset was split into calibration and validation subsets using a 5-fold cross-validation with 100 repetitions. Different choices of folds and repetitions yielded similar results. Second, for each repetition, the calibration subset was used to build a multiple linear regression between the cost factor $c_{i}$ at site i, and their corresponding site-specific environmental variables and leaf traits,$\;x_{ij}$, depending on the scenario:

(7) $$c_{i}=\beta_{0}+\sum_{j}\beta_{j}x_{ij}$$

where $\beta_{j}$ are fitted parameters for the specific environmental variable or leaf trait (see the example demonstration in Table S4 and S5). The multiple linear regression coefficients of predictors were fitted using a genetic algorithm (GA) technique through minimizing the RMSE between observed and optimality-dynamic modelled *V*_c, maxTg_. GA is a heuristic global optimization method [47] that avoids the dependence on the initial parameter values and efficiently identifies the global parameter optimization solution when there are many acceptable local solutions [48]. Third, for each repetition, we used the multiple linear regression coefficients estimated from the calibration sub-dataset to predict *V*_c, max_ of the independent validation sub-dataset. Fourth, the predicted *V*_c, max_ was then averaged across all 100 repetitions to obtain the ensemble predicted value for each measurement record, which was used to evaluate the optimality model performance under each scenario.

### 2.4 Cross-model performance comparisons and attributions of important variables of the two modelling approaches

With the above model-derived *V*_c, maxTg_ and *V*_c, max25_, we evaluated the model performance with reference to field-derived *V*_c, maxTg_ and *V*_c, max25_. Four statistical metrics for assessing model performance include (1) *r*^2^–the square of correlation coefficient, (2) Bias–the residual bias, (3) RMSE, and (4) AIC–Akaike Information Criterion. The AIC was calculated with the formula AIC = 2 × *k* + *n* × log (RMSE^2^), where *n* is the number of observations, and *k* is the number of model parameters.

For the statistical modelling approach, we assessed the relative importance of each environmental or biotic variable on *V*_c, max25_ modelling. To avoid the strong multicollinearity amongst predictor variables (Fig. S7), we first computed the variance inflation factor (VIF) and iteratively removed variables with very high VIF [49], [50], until the remaining variables with VIF less than 10 and the absolute values of correlation coefficients amongst these variables were less than 0.7. Afterwards, we conducted a model selection for *V*_c, max25_ based on corrected AIC using ‘glmulti’ *R* package [51]. We estimated the relative importance of each variable as the sum of the Akaike weights for the models in which the variable appeared. We set relative importance value of 0.8 as a cut-off to differentiate between important and unimportant variables [52]. Finally, we illustrated the sign of the effects from each selected variable through conducting partial regression plots while holding all the other variables constant [51,53].

For the optimality-constant model, we used a sensitivity analysis to assess the variable importance. Specifically, we partitioned the variance of the key model output into the variation in each model input (i.e., *T*_g_, PAR, VPD, elevation, and *C*_a_), allowing these inputs to cover the full range of environmental variables. The analysis includes three steps. First was the set-up of the five environmental variables. We built a one-time random sampling for each of the five environmental variables using Sobol’ quasi-random sequences [54]. Second were the model simulations of *V*_c, max25_ using the optimality-constant model, with the five environmental variables as input. Third was the variance partitioning. We employed a global variance-based sensitivity analysis algorithm developed by Saltelli et al. [55], and partitioned the variance of modelled *V*_c, max25_ driven by each environmental variable, by which we quantified the variables’ relative importance.

## 3 Results

### 3.1 Relative importance of enzyme kinetics and eco-evo-environmental drivers of *V*_c, max25_ in explaining global *V*_c, maxTg_ variability

**Fig. 1:**
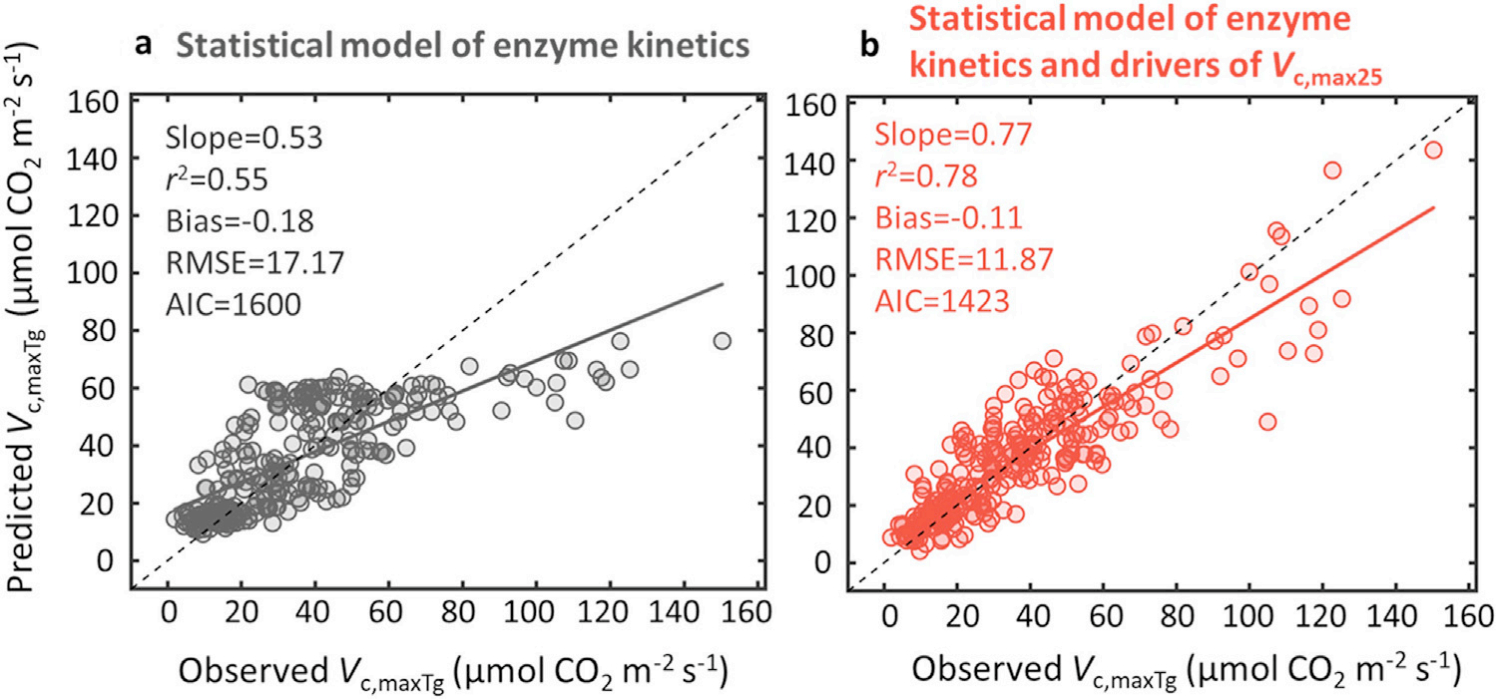
**The relative importance of enzyme kinetics and eco-evo-environmental drivers of *V*_c, max25_ in controlling global *V*_c, maxTg_ variability.** The statistical modelling approach is analyzed at two levels: global *V*_c, maxTg_ variability is described using (a) the temperature-associated enzyme kinetics together with a globally averaged *V*_c, max25_, or (b) by adding temperature-associated enzyme kinetics to site-specific *V*_c, max25_ derived from its empirical relationship with both environmental variables and leaf traits. Details of these two models with the fitted equations are presented in Tables S5 and S6. Four statistical metrics for assessing model performance include (1) *r*^2^–the square of correlation coefficient, (2) Bias–the residual bias, (3) RMSE–the root mean square of error, and (4) AIC–Akaike Information Criterion. Lines are fitted by the ordinary least-square regressions.

To investigate the relative importance of enzyme kinetics and eco-evo-environmental drivers of *V*_c, max25_ in explaining global *V*_c, maxTg_ variability, we analyzed the relationships between field-derived and model-predicted *V*_c, maxTg_ using the statistical modelling approach. We found that the statistical approach considering both factors explained 78% of global *V*_c, maxTg_ variability (RMSE = 11.87 µmol CO_2_ m^−2^ s^−1^, AIC = 1423; Fig. 1b), followed by 55% explained by enzyme kinetics alone (RMSE = 17.17 µmol CO_2_ m^−2^ s^−1^; AIC = 1600; Fig. 1a). This result demonstrates that temperature-associated enzyme kinetics dominate the explanation of global *V*_c, maxTg_ variability, with eco-evo-environmental drivers of *V*_c, max25_ also exerting a considerable role.

### 3.2 The performance of optimality-constant model and the reasons underlying its poorer performance

**Fig. 2:**
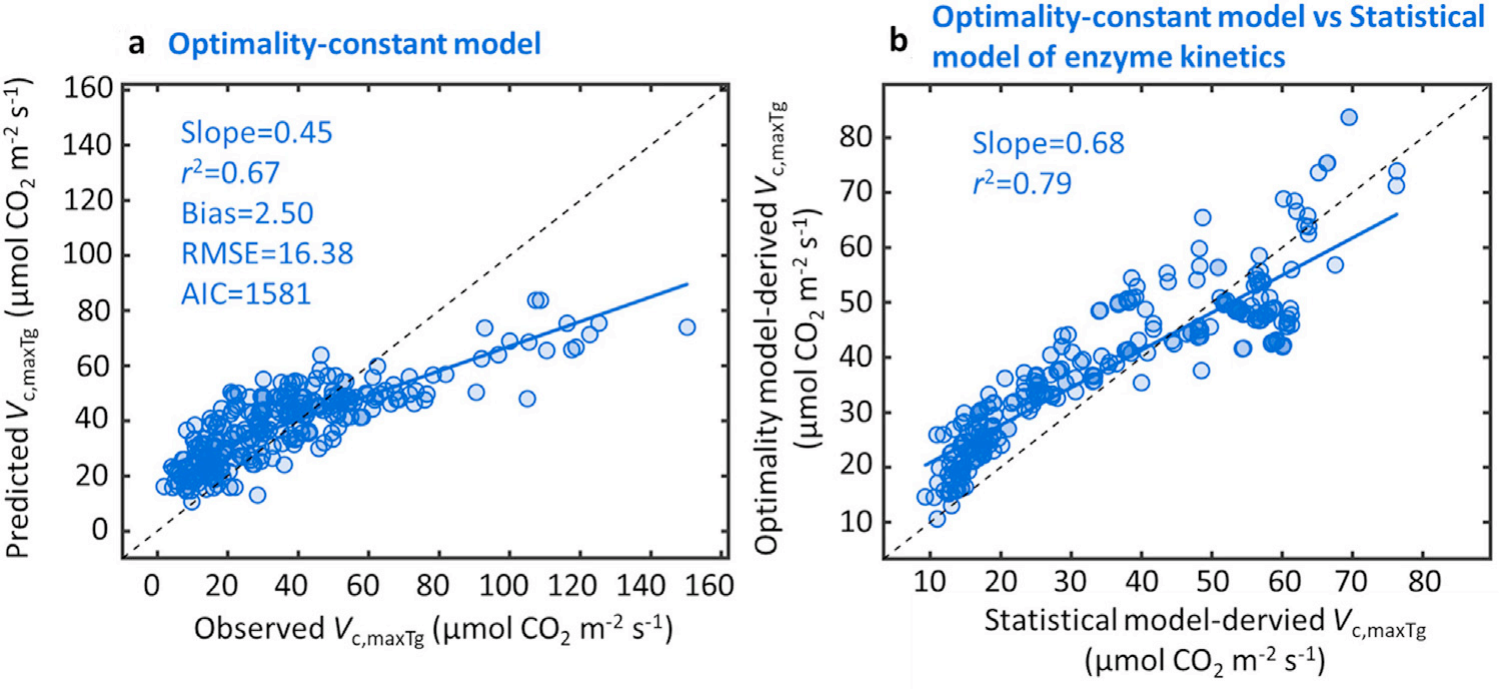
**The performance of optimality-constant model in predicting global *V*_c, maxTg_ variability.** The optimality model is used for global *V*_c, maxTg_ prediction with a default globally constant *c* (i.e., the total unit carbon cost of building and maintaining the photosynthetic machinery), or the optimality-constant model (a), which displays high correlation as the statistical model of enzyme kinetics (b). The statistical model of enzyme kinetics here is the same as Fig. 1a.

**Fig. 3:**
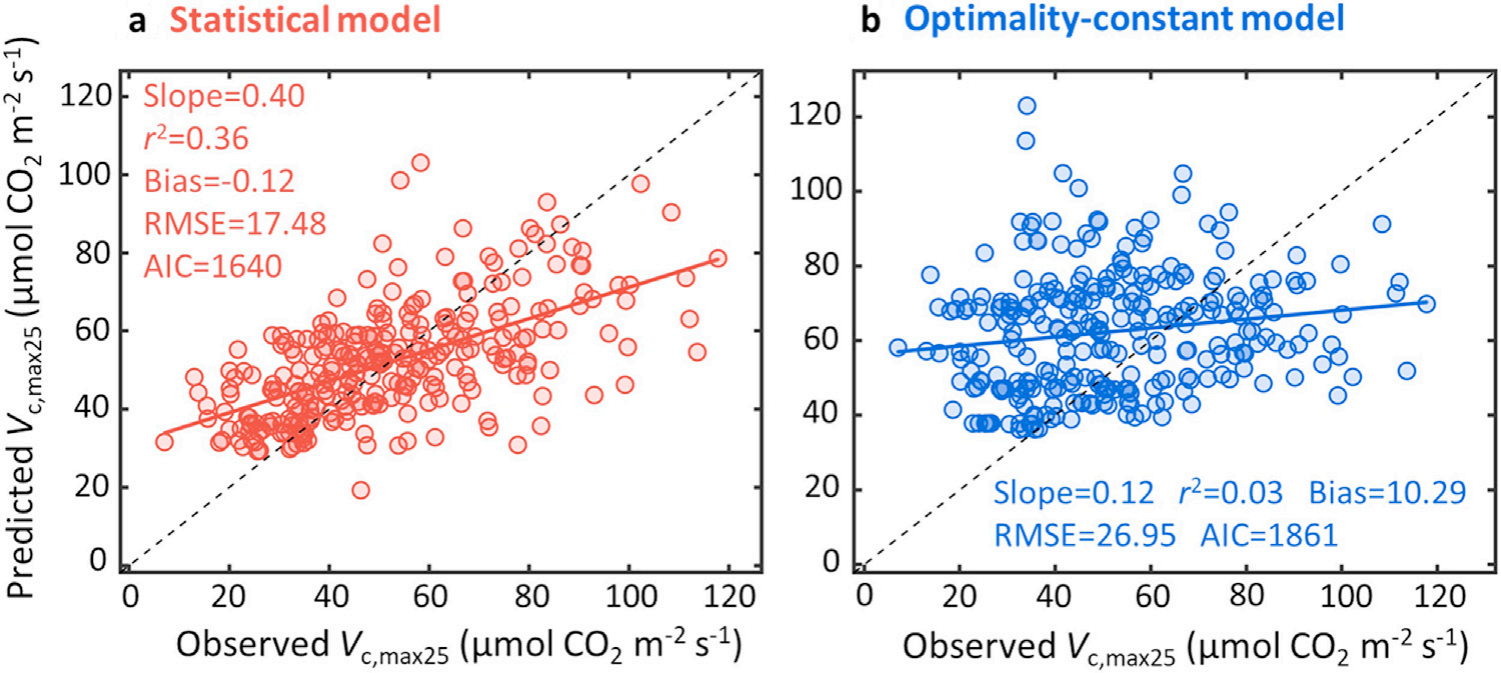
**The statistical model of environmental variables and leaf traits outperforms the optimality-constant model in predicting *V*_c, max25_.** The comparison of field-derived *V*_c, max25_ with (a) the statistical model derived *V*_c, max25_ that relies on its multiple linear regression relationship with both environmental variables and leaf traits, and (b) the *V*_c, max25_ derived from the optimality-constant model. Details of the statistical model with the fitted equations are presented in Tables S4 and S5.

To investigate whether optimality-constant model explains global pattern of *V*_c, max_, we cross-compared the optimality-constant model with the statistical model. We found that the optimality-constant model explained 67% of global *V*_c, maxTg_ variability but with comparable model error (RMSE = 16.38 µmol CO_2_ m^−2^ s^−1^; AIC = 1581; Fig. 2a) as the statistical model considering enzyme kinetics alone (RMSE = 17.17 µmol CO_2_ m^−2^ s^−1^; AIC = 1600; Fig. 1a). We further found that the predicted *V*_c, maxTg_ by optimality-constant model was highly correlated with that predicted using the statistical model considering enzyme kinetics alone (*r*^2^ = 0.79; Fig. 2b), suggesting that the optimality-constant model likely captured similar ecophysiological processes as the enzyme kinetics’ response, and thus showed comparable model performance in predicting *V*_c, maxTg_ (Fig. 1a vs Fig. 2a). This also agreed with the *V*_c, max25_ modelling result, as environmental variables and leaf traits altogether explained 36% of *V*_c, max25_ variability (Fig. 3a; Table S1), but optimality-constant model explained only 3% of *V*_c, max25_ variability (Fig. 3b).

**Fig. 4:**
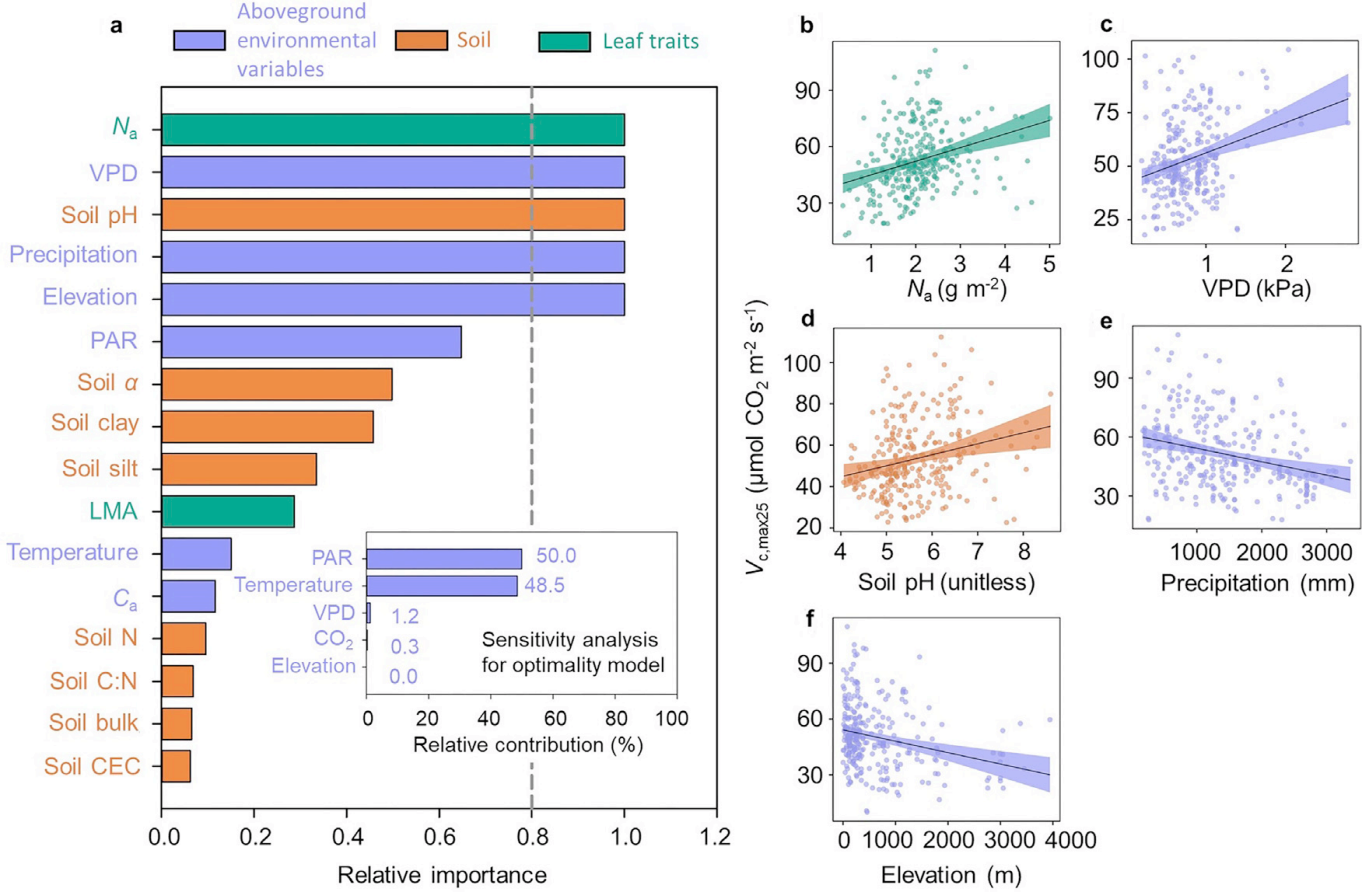
**Relative importance of environmental variables and leaf traits in predicting global *V*_c, max25_ variability.** (a-main) The relative importance of each variable based on the sum of the Akaike weights derived from a model selection using corrected AIC; (a-inset) the relative contribution of the five aboveground environmental variables to *V*_c, max25_ based on the sensitivity analysis of the optimality-constant model; (b-f) partial regression plots of *V*_c, max25_ with the predictor of area-based leaf nitrogen content (*N*_a_), vapor pressure deficit (VPD), soil pH, precipitation, and elevation, respectively. The cutoff (dashed line) of panel (a) is set at 0.8 for identifying the most important predictor variables; the shade areas in (b-f) are 95% confidential intervals around the predicted relationships.

To explore the reasons underlying the poorer performance of optimality-constant model in predicting *V*_c, max25_ variability further, we cross-compared the relative importance of each variable that was represented in the optimality-constant and statistical modelling of *V*_c, max25_. Based on the statistical modelling approach, we identified five most important biotic and environmental variables for explaining *V*_c, max25_ variability, i.e., *N*_a_, VPD, soil pH, precipitation and elevation, following a descending order of their relative importance (Fig. 4a). This result was different from the optimality-constant model, in which PAR and temperature were identified as the two most important variables (with relative contributions of 50.0% and 48.5%, respectively) for *V*_c, max25_ (Fig. 4a). Collectively, these results demonstrate that the important drivers of global *V*_c, max25_ (and thus *V*_c, maxTg_) variability were not well represented in optimality-constant model.

### 3.3 The improvement of optimality model through considering belowground resource constraints

**Fig. 5:**
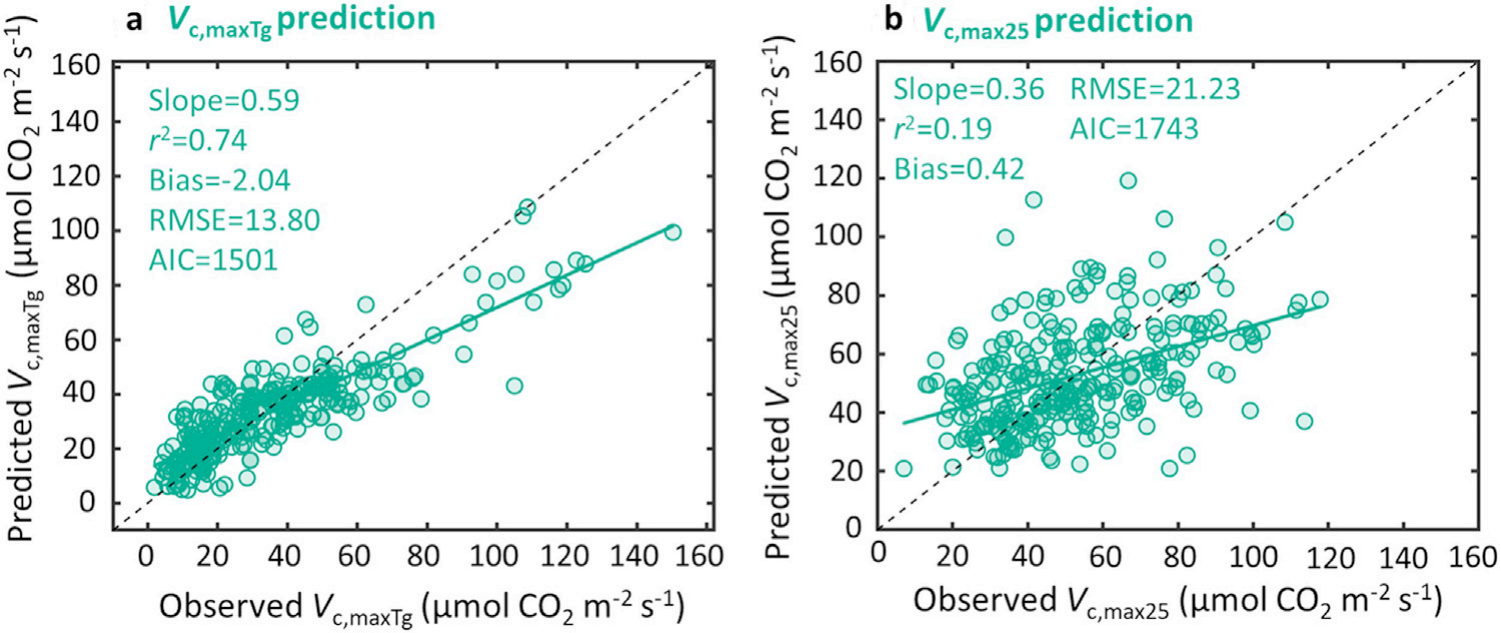
**The performance of optimality-dynamic model in predicting global variability of *V*_c, maxTg_ (a) and *V*_c, max25_ (b).** The optimality model is used for global *V*_c, maxTg_ and *V*_c, max25_ predictions with a site-specific dynamic *c* (i.e., the total unit carbon cost of building and maintaining the photosynthetic machinery) constrained by edaphic variables, or the optimality-dynamic model. Details of the optimality-dynamic model with the fitted equations for cost factor *c* parameterization are presented in Tables S4 and S5.

To investigate whether including belowground resource constraints on photosynthetic machinery would improve current optimality modelling of global *V*_c, max_ variability, we revised the current optimality model by including a dynamic representation of *c* that described belowground resource constraints through edaphic variables. We found that optimality-dynamic model had a much-improved predictive power and reduced model error for *V*_c, maxTg_ variability (*r*^2^ = 0.74; RMSE = 13.80 µmol CO_2_ m^−2^ s^−1^; AIC = 1501; Fig. 5a), but still showed a slightly subordinate performance compared with the statistical model of both enzyme kinetics and eco-evo-environmental drivers of *V*_c, max25_ (Fig. 1b). We also observed that optimality-dynamic model explained 19% of *V*_c, max25_ variability (Fig. 5b), which was larger than that (3%) explained by optimality-constant model (Fig. 3b). Such a cross-comparison further demonstrates that the improvement in modelling *V*_c, max25_ through considering belowground resource constraints on *c* indeed represents a key avenue for improved modelling of global *V*_c, max_ variability.

## 4 Discussion

This study has three main findings. First, the statistical modelling approach considering enzyme kinetics and eco-evo-environmental drivers of *V*_c, max25_ explained a large portion (78%) of global *V*_c, maxTg_ variability, with enzyme kinetics as the dominant factor explaining 55% of global *V*_c, maxTg_ variability. Second, treating the statistical model as a benchmark for the optimality approach to predict *V*_c, maxTg_, we found that the optimality-constant model effectively captured the role of enzyme kinetics in controlling global *V*_c, maxTg_ variability, but was ineffective in capturing the second control on global *V*_c, maxTg_ variability through eco-evo-environmental drivers of *V*_c, max25_. Third, we found that belowground resources added constraints on building and maintaining photosynthetic machinery, and improved the modelling of *V*_c, max25_ variability.

### 4.1 Enzyme kinetics and eco-evo-environmental drivers of *V*_c, max25_ jointly determine global *V*_c, maxTg_ variability

Our results first showed that enzyme kinetics’ response was the dominant regulator of global *V*_c, maxTg_ variability (Fig. 1). One possible explanation for this is the thermal sensitivity of the biochemical components involved in the RuBisCO enzyme kinetics [56], [57], and thus the temperature-associated enzyme kinetics exert a direct, positive control on global *V*_c, maxTg_ variability. To further elucidate the role of enzyme kinetics, we examined the empirical relationship between *V*_c, maxTg_ and both environmental variables and leaf traits. Our analysis revealed that these predictors together accounted for 71% of *V*_c, maxTg_ variability (Fig. S8a), with growing-season temperature being the dominant predictor (55%) (Fig. S8b), which is equivalent to the explanatory capacity to the enzyme kinetics statistical model (Fig. 1a). We also investigated the relative importance of each variable in predicting *V*_c, maxTg_ variability, and identified the four most biotic and environmental variables, namely *T*_g_, VPD, *N*_a_, and elevation, in descending order of their relative importance (Fig. S9). These results collectively consolidate the dominant role of enzyme kinetics in global *V*_c, maxTg_ variability.

Interestingly, several previous studies have reported weak, yet significant, negative relationships between *V*_c, max25_ and site-mean growing temperature across global vegetative landscapes [6,^29^,58]. However, utilizing the largest global *V*_c, max_ dataset compiled in this study, we found that the bivariate ordinary least-squares regression relationship between *T*_g_ and *V*_c, max25_ was insignificant (Fig. S7). The key factors for explaining *V*_c, max25_ variability, based on the sum of the Akaike weights, included leaf N content, VPD, soil pH, precipitation, and elevation, but not the *T*_g_ factor (Fig. 4). The minimal effect of *T*_g_ on *V*_c, max25_ variability is also supported by another similar study that explored the best model of global *V*_c, max25_ variability [62]. The potential cause for *T*_g_’s minimal effect might be related to the interactive effects among multiple interrelated environmental variables at a global scale (Fig. S7), with the apparent temperature effect being hidden by other associated variables, such as VPD, soil pH and precipitation (Fig. 4c-e). These results together suggest that such a temperature-induced *V*_c, max25_ down-regulation is not sufficient to compensate the direct, positive effect of temperature on *V*_c, maxTg_ through enzyme kinetics’ response, and thus the positive temperature-*V*_c, maxTg_ relationship is observed on a global scale. Collectively, our results imply that temperature-associated enzyme kinetics represent the first-order control on the variability of *V*_c, maxTg_, which would increase with warmer growing season temperatures.

Second, we found that eco-evo-environmental drivers of *V*_c, max25_ also importantly regulated global *V*_c, maxTg_ variability (Fig. 1b). Thus, when we added the site-specific *V*_c, max25_ to the temperature-associated enzyme kinetics, the explained variance of global *V*_c, maxTg_ variability increased from 55% to 78%, even though the environmental variables and leaf traits altogether only explained 36% of *V*_c, max25_ variability. These results altogether further generate two important implications: (1) improved characterization of *V*_c, max25_ variability represents a very effective avenue for modelling global *V*_c, maxTg_ variability, and (2) there remains a large proportion of *V*_c, max25_ variability unexplained. Some of the unexplained *V*_c, max25_ variability may be attributed to random measuring and sampling error in the evaluation of site-mean level *V*_c, max25_ [41]. Alternatively, other important environmental factors (e.g., day length, soil moisture, and soil phosphorus (P) content) [6,^29^,37], unmeasured leaf traits (e.g., leaf P content, and leaf age) [3,59], species properties [29,30], and evolutionary history [60] might have also played important roles in driving *V*_c, max25_ variability.

### 4.2 Limitations of optimality-constant model in capturing global *V*_c, maxTg_ variability

There is an increasing interest in using the optimality-constant model for characterizing *V*_c, max25_ variability for different applications, such as exploring the patterns of *V*_c, max25_ variation across environmental gradients and parameterizing TBMs [12,18]. However, our study revealed that optimality-constant model performed relatively worse than the optimality-dynamic model in predicting global *V*_c, maxTg_ variability, which was primarily attributed to the low predictive power (*r*^2^ = 0.03) of the global *V*_c, max25_ (Fig. 3b). Our findings thus highlight that optimality-constant model development should focus on improvements in capturing large-scale *V*_c, max25_ variability. Notably, Peng et al. (2021) using the similar global dataset, reported a much higher *V*_c, max25_ prediction (i.e. *r*^2^ = 0.36) based on the optimality model [12], which is different from ours (*r*^2^ = 0.03). Our further analysis suggested that this discrepancy is primarily linked to the inaccurate dataset (ultilizing the mean growing season temperature to approximate *V*_c, max_ records without direct leaf temperature measurements) employed in Peng et al. (2021)’s data analysis [12]. Upon removing this inaccurate dataset (i.e. the *V*_c, max_ records transformed from the mean growing season temperature) from the data analysis, the result comparable to ours (*r*^2^ = 0.03) was then found (Fig. S2b). Such cross-comparisons also suggest that our analysis is rigorous and the generated result is trustworthy.

Our results also help identify that the poorer performance of optimality-constant model was mainly because of its failure to capture the dominant environmental and biotic regulations of global *V*_c, max25_ variability, including *N*_a_, VPD, soil pH, precipitation, and elevation (Fig. 4). In contrast, optimality-constant model only included five aboveground environmental variables (i.e., *T_g_*, VPD, PAR, *C_a_* and elevation), with PAR and *T*_g_ alone capturing 98.5% of *V*_c, max25_ variability in model predictions (Fig. 4a). Our results suggested that other variables, including belowground resource availability, also importantly regulated *V*_c, max25_ variability (Fig. 4b-f). For example, *N*_a_ may reflect soil N availability [61] and has been proven to be an essential element for the construction of RuBisCO and other essential enzymes of the photosynthetic machinery and thus, *N*_a_ positively correlates with *V*_c, max25_ [3,10]. Soil pH is a positive indicator of soil fertility, and higher soil pH usually means a lower nutrient cost for the construction of RuBisCO, thus favoring plant investment in greater *V*_c, max25_ [27,37]. VPD and precipitation reflect the water stress status that importantly mediates plant stomatal behaviors, and high VPD and low precipitation generally reduce stomatal conductance and then increase the investment in *V*_c, max25_ required to achieve optimal photosynthesis [13,62]. However, the relationship between soil pH and soil fertility is non-linear and dependent on plant species types [66], leading to a peak in *V*_c, max25_ at intermediate soil pH levels (Fig. 4d). Similarly, the relationship between VPD and *V*_c, max25_ exhibits non-linear and monotonic trends, rather than linear ones (Fig. 4c). These findings indicate that the use of linear regressions to derive explanatory variables for *V*_c, max25_ has certain limitations, and future studies should consider non-linear explanations for key factors influencing *V*_c, max25_ variability..

We also observed a negative effect of elevation alone on *V*_c, max25_ (Fig. 4f), which contradicts previous findings that higher *V*_c, max25_ is necessary to maximize carbon assimilation at high elevations when examining specific elevation transects [19,^25^,42]. This discrepancy may be attributed to complex and interactive effects of numerous interrelated environmental variables and leaf traits across large geographical extents (Fig. S5). As a result, the direct effect of elevation on *V*_c, max25_ differs from the apparent effects often confounded by other interrelated variables, including *N*_a_, VPD, soil pH and precipitation (Fig. 4b-f). Collectively, these explored dominant environmental and biotic variables further consolidate important drivers associated with global *V*_c, max25_ (and thus *V*_c, maxTg_) variability, which are not comprehensively represented in current optimality-constant model.

It is important to note that, besides soil nutrient availability, atmospheric N deposition also serves as a significant source of plant N uptake and demand, potentially affecting plant N concentration and subsequently *V*_c, max25_ [10,^67^,68]. The magnitude of N deposition has fluctuated significantly over the time across the past decades [67,68]. However, our study lacks the necessary sampling year information to match global *V*_c, max_ observations at a temporal scale, making it difficult to incorporate atmospheric N deposition into the analysis of factors driving global *V*_c, max25_ variability. Further manipulative experiments and field monitoring are warranted to disentangle the relative importance of atmospheric N deposition and soil N availability in regulating the amount of RuBisCO enzyme and, subsequently, *V*_c, max25_ variability. Besides, our study primarily examined the individual role of each environmental variable and leaf trait in driving global *V*_c, max25_ variability, as previously mentioned. However, our findings also revealed the combined influence of climate variables, soil properties, and leaf traits on *V*_c, max25_ variability (Fig. S10), suggesting that belowground properties can regulate *V*_c, max25_ variability by indirectly affecting aboveground plant performance, while aboveground climate properties and plant traits can also influence *V*_c, max25_ variability through indirect changes to belowground chemical and physical properties. Thus, to fully understand the intrinsic mechanisms of global *V*_c, max25_ variability, both independent and interactive effects of explanatory variables must be thoroughly considered.

### 4.3 Considering the belowground resource constraints can improve the performance of optimality model

Our results showed that optimality-dynamic model considering the belowground resource constraints outperformed optimality-constant model in predicting both *V*_c, maxTg_ and *V*_c, max25_. This finding is consistent with recent studies, which suggest that edaphic variables can modify leaf economics [27,37]. The explanation is that edaphic variables influence the availability of soil nutrients and water and, consequently, the uptake costs for the construction and maintenance of photosynthetic machinery [27,37]. For example, a large fraction of leaf N (∼50%) is allocated to the photosynthetic apparatus [63]. The cost of root N uptake varies with soil N availability and N acquisition strategies [37,64]. Similarly, soil water content influences the water uptake and transport costs necessary to deliver water to the leaves, which can affect the investments to support assimilation [13,27]. These considerations thus imply that the cost of belowground resource uptake and use could be a first-order priority for improving theoretical modelling of global *V*_c, max_ variability.

**Fig. 6:**
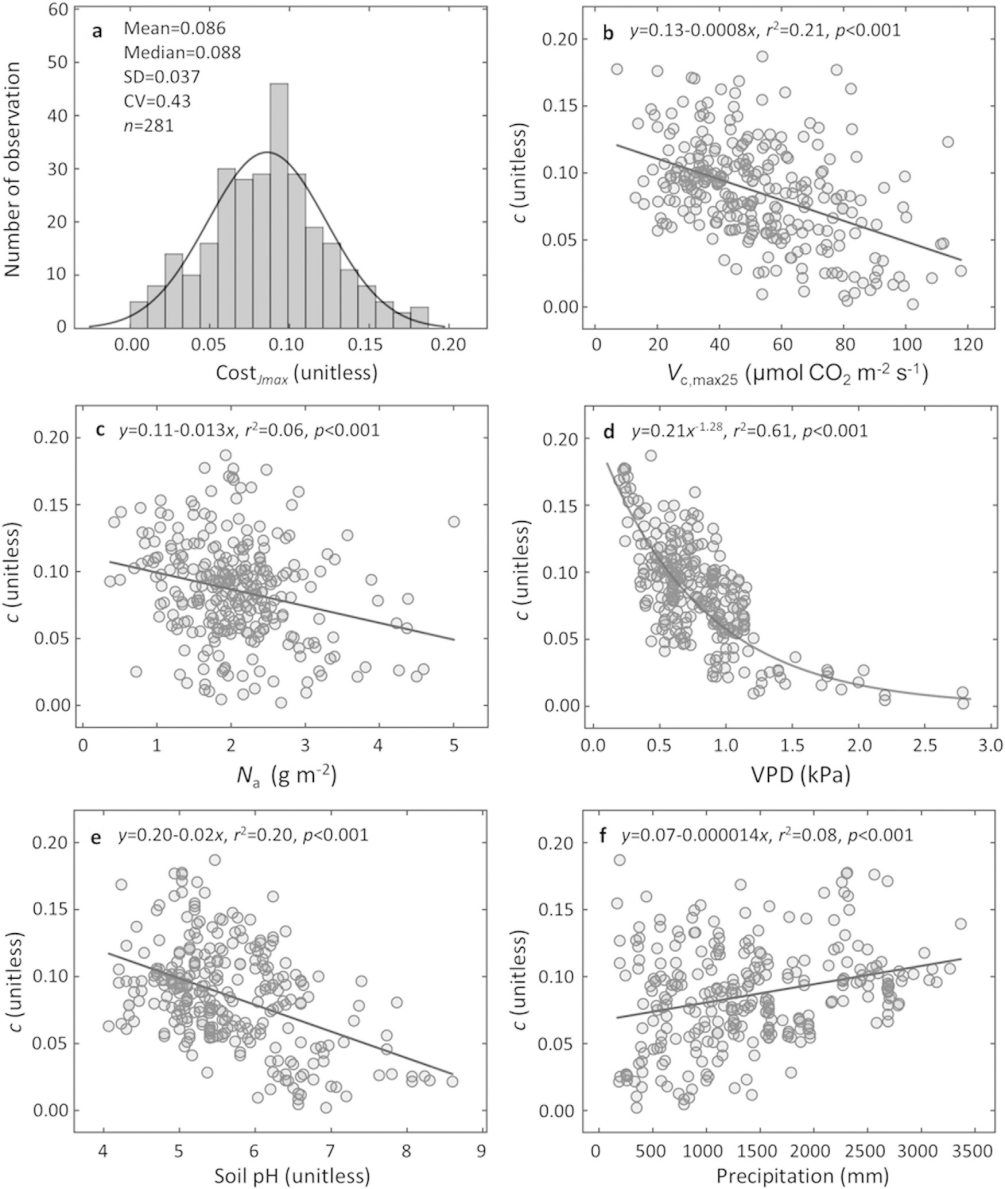
**The cost factor *c* constrained by edaphic variables shows large variability and is highly related to environmental variables and leaf traits.** (a) The histogram distribution of the *c* across the global 281 sites. The bolded black line indicates the probability distribution function used to fit the histogram distribution; four statistical metrics are used to indicate the characteristics of *c* distribution, including mean, median, standard deviation (SD) and coefficient of variation (CV). (b-f) The ordinary least-square regression plots of *c* with the predictor of *V*_c, max25_, area-based leaf nitrogen content (*N*_a_), vapor pressure deficit (VPD), soil pH, and precipitation, respectively. *r*^2^ and *p*-value represent the square of correlation coefficient and significance level, respectively.

In addition, we tested the environmental constraints on parameter *c* through two other scenarios, i.e., the five most important variables revealed by the statistical model (Fig. 4), and all the environmental and biotic variables. Overall, these three scenarios achieved very comparable results in predicting both *V*_c, maxTg_ (Table S2) and *V*_c, max25_ (Table S3), with the *r*^2^ of 0.74-0.75 and RMSE of 13.51-13.80 for *V*_c, maxTg_, and *r*^2^ of 0.19-0.22 and RMSE of 19.64-21.63 for *V*_c, max25_, respectively. These further lend us confidence in the following three interpretations: (1) our empirically identified five most important variables for *V*_c, max25_ (Fig. 4) are ecologically meaningful, (2) these environmental and biotic variables could shape the *V*_c, max25_ biogeography through regulating the site-specific variability in *c* (Fig. 6b-f), and (3) the optimality model when considering a variable *c* indeed improves the modelling of both *V*_c, max25_ and *V*_c, maxTg_.

The trends of *c* with environmental and biotic variables also agree with the idea that allocation costs vary with environmental gradients (Fig. 6b-f). Parameter *c* represents the total unit cost of constructing and maintaining the photosynthetic apparatus. Previous empirical studies have demonstrated that the construction cost of photosynthetic apparatus is influenced by belowground resource availability [26,27]. The proportion of leaf N allocated to the RuBisCO enzyme and the cost of root N uptake are influenced by soil nutrient and water availability, as well as associated N acquisition strategies [13,^27^,^37^,64]. Our results reveal that the cost factor *c* exhibits considerable variability and is influenced by edaphic properties. We identified a significantly negative relationship between parameter *c* and soil pH, indicating decreasing carbon costs at high soil pH levels with greater nutrient availability. This observation is supported by increased N allocation to the RuBisCO enzyme at higher pH levels [62] and lower leaf respiration and nutrient acquisition costs on alkaline, more fertile soils [27,65]. Furthermore, we discovered a significantly positive relationship between parameter *c* and soil *α* (a positive indicator of plant-available surface moisture) (results not shown), suggesting the decreasing carbon costs under drier conditions, accompanied by higher N allocation to the RuBisCO enzyme and subsequently, *V*_c, max25_ [13,27].The parameterization of the cost functions allows including important environmental variables in a more mechanistic framework than the statistical regression modelling. However, our current parametrization of *c* is purely phenomenological. More research will be needed to understand how the cost functions relate to the biological processes of building and maintaining the photosynthetic machinery.

### 4.4 Implication and future directions

Our work generates two implications and future directions for understanding controls of global *V*_c, maxTg_ variability and terrestrial biosphere modelling. First, the proposed statistical modelling approach captures the synthetic effects of enzyme kinetics, environmental variables and leaf traits on *V*_c, maxTg_, providing an improved understanding of global *V*_c, maxTg_ variability. The accurate prediction of *V*_c, maxTg_ by considering both factors of enzyme kinetics and eco-evo-environmental drivers of *V*_c, max25_ highlights that there are timescale-dependent mechanisms in regulating global *V*_c, maxTg_ variability, with enzyme kinetics capturing instantaneous ecophysiological responses and explaining the dominant variance of global *V*_c, maxTg_. Meanwhile, the second factor related to environmental variables and leaf traits likely represents the eco-evolutionary control of *V*_c, maxTg_ through structuring *V*_c, max25_ biogeography. Although the second factor is empirical, we further hypothesize that this might be associated with several key eco-evolutionary processes, e.g., abiotic filtering of species pools, competition for limited resources and the resultant trait-trait relationships subject to fundamental evolutionary principles [23,^30^,^31^,60]. However, further rigorous hypothesis testing related to eco-evolutionary processes of *V*_c, max25_ through experimental manipulation and field observation approaches across large environmental gradients is still needed.

Second, the two factors that both regulate global *V*_c, maxTg_ variability also provide an important benchmark and theoretical basis for evaluating current optimality models [8,19] while inspiring future improvements in the model representation of *V*_c, maxTg_. For example, our results highlight that optimality-constant model may struggle primarily to capture *V*_c, max25_ variability (Fig. 3b), probably due to an incomplete representation of environmental and biotic regulations on the cost functions. These findings suggest that these deficiencies should be addressed to more reliably model photosynthetic processes in TBMs [5,18]. To achieve this, sophisticated observational and experimental studies are still needed to help elucidate the reasons for the near-zero predictability of *V*_c, max25_ in current optimality-constant model, as well as exploring potential ways to mechanistically improve optimality modelling, and thus better constrain TBMs for improved representation of terrestrial photosynthesis, carbon cycling and climate change [1], [2].

## Data availability statement

All data used in our study will be uploaded to the Dryad Digital Repository after the manuscript is accepted.

## CRediT authorship contribution statement

**Zhengbing Yan:** Conceptualization, Data curation, Formal analysis, Investigation, Methodology, Project administration, Resources, Software, Validation, Visualization, Writing – original draft, Writing – review & editing. **Matteo Detto:** Methodology, Supervision, Writing – review & editing. **Zhengfei Guo:** Methodology, Writing – review & editing. **Nicholas G. Smith:** Writing – review & editing. **Han Wang:** Writing – review & editing. **Loren P. Albert:** Writing – review & editing. **Xiangtao Xu:** Writing – review & editing. **Ziyu Lin:** Methodology. **Shuwen Liu:** Methodology. **Yingyi Zhao:** Methodology. **Shuli Chen:** Methodology. **Timothy C. Bonebrake:** Writing – review & editing. **Jin Wu:** Conceptualization, Formal analysis, Funding acquisition, Investigation, Methodology, Project administration, Supervision, Writing – original draft, Writing – review & editing.

## Appendix Supplementary materials

Image, application 1

## References

[1] Bonan G.B., Doney S.C. (2018). Climate, ecosystems, and planetary futures: The challenge to predict life in Earth system models. Science.

[2] Ryu Y., Berry J.A., Baldocchi D.D. (2019). What is global photosynthesis? History, uncertainties and opportunities. Remote Sens. Environ..

[3] Walker A.P., Beckerman A.P., Gu L.H. (2014). The relationship of leaf photosynthetic traits-*V*_cmax_ and *J*_max_ to leaf nitrogen, leaf phosphorus, and specific leaf area: A meta-analysis and modeling study. Ecol. Evol..

[4] Farquhar G.V., von Caemmerer S.V., Berry J.A. (1980). A biochemical model of photosynthetic CO_2_ assimilation in leaves of C_3_ species. Planta.

[5] Rogers A., Medlyn B.E., Dukes J.S. (2017). A roadmap for improving the representation of photosynthesis in Earth system models. New Phytol..

[6] Ali A.A., Xu C.G., Rogers A. (2015). Global-scale environmental control of plant photosynthetic capacity. Ecol. Appl..

[7] Croft H., Chen J.M., Luo X.Z. (2017). Leaf chlorophyll content as a proxy for leaf photosynthetic capacity. Glob. Change Biol..

[8] Smith N.G., Keenan T.F., Prentice I.C. (2019). Global photosynthetic capacity is optimized to the environment. Ecol. Lett..

[9] Qian X., Liu L., Croft H. (2021). Relationship between leaf maximum carboxylation rate and chlorophyll content preserved across 13 species. J. Geophys. Res.-Biogeosci..

[10] Kattge J., Knorr W., Raddatz T. (2009). Quantifying photosynthetic capacity and its relationship to leaf nitrogen content for global-scale terrestrial biosphere models. Glob. Change Biol..

[11] Ali A.A., Xu C.G., Rogers A. (2016). A global scale mechanistic model of photosynthetic capacity (LUNA V1. 0). Geosci. Model Dev..

[12] Peng Y.K., Bloomfield K.J., Cernusak L.A. (2021). Global climate and nutrient controls of photosynthetic capacity. Commun. Biol..

[13] Prentice I.C., Dong N., Gleason S.M. (2014). Balancing the costs of carbon gain and water transport: Testing a new theoretical framework for plant functional ecology. Ecol. Lett..

[14] Norby R.J., Gu L.H., Haworth I.C. (2017). Informing models through empirical relationships between foliar phosphorus, nitrogen and photosynthesis across diverse woody species in tropical forests of Panama. New Phytol..

[15] Detto M., Xu X. (2020). Optimal leaf life strategies determine *V*_c,max_ dynamic during ontogeny. New Phytol..

[16] Yan Z.B., Guo Z.F., Serbin S.P. (2021). Spectroscopy outperforms leaf trait relationships for predicting photosynthetic capacity across different forest types. New Phytol..

[17] Wang H., Atkin O.K., Keenan T.F. (2020). Acclimation of leaf respiration consistent with optimal photosynthetic capacity. Glob. Change Biol..

[18] Luo X.Z., Keenan T.F. (2020). Global evidence for the acclimation of ecosystem photosynthesis to light. Nat. Ecol. Evol..

[19] Wang H., Prentice I.C., Keenan T.F. (2017). Towards a universal model for carbon dioxide uptake by plants. Nat. Plants.

[20] Wang H., Prentice I.C., Wright I.J. (2023). Leaf economics fundamentals explained by optimality principles. Sci. Adv..

[21] Dong N., Prentice I.C., Wright I.J. (2022). Leaf nitrogen from the perspective of optimal plant function. J. Ecol..

[22] Wright I.J., Reich P.B., Westoby M. (2003). Least-cost input mixtures of water and nitrogen for photosynthesis. Am. Nat..

[23] Harrison S.P., Cramer W., Franklin O. (2021). Eco-evolutionary optimality as a means to improve vegetation and land-surface models. New Phytol..

[24] Smith N.G., Keenan T.F. (2020). Mechanisms underlying leaf photosynthetic acclimation to warming and elevated CO_2_ as inferred from least-cost optimality theory. Glob. Change Biol..

[25] Peng Y.K., Bloomfield K.J., Prentice I.C. (2020). A theory of plant function helps to explain leaf-trait and productivity responses to elevation. New Phytol..

[26] Bahar N.H.A., Ishida F.Y., Weerasinghe L.K. (2017). Leaf-level photosynthetic capacity in lowland Amazonian and high-elevation Andean tropical moist forests of Peru. New Phytol..

[27] Paillassa J., Wright I.J., Prentice I.C. (2020). When and where soil is important to modify the carbon and water economy of leaves. New Phytol..

[28] Kattge J., Knorr W. (2007). Temperature acclimation in a biochemical model of photosynthesis: A reanalysis of data from 36 species. Plant Cell Environ..

[29] Smith N.G., Dukes J.S. (2018). Drivers of leaf carbon exchange capacity across biomes at the continental scale. Ecology.

[30] Wright I.J., Reich P.B., Westoby M. (2004). The worldwide leaf economics spectrum. Nature.

[31] Reich P.B. (2014). The world-wide ‘fast–slow’ plant economics spectrum: A traits manifesto. J. Ecol..

[32] Meir P., Shenkin A., Disney M. (2017).

[33] Domingues T.F., Meir P., Feldpausch T.R. (2010). Co-limitation of photosynthetic capacity by nitrogen and phosphorus in West Africa woodlands. Plant Cell Environ..

[34] Domingues T.F., Ishida F.Y., Feldpausch T.R. (2015). Biome-specific effects of nitrogen and phosphorus on the photosynthetic characteristics of trees at a forest-savanna boundary in Cameroon. Oecologia.

[35] Cernusak L.A., Hutley L.B., Beringer J. (2011). Photosynthetic physiology of eucalypts along a sub-continental rainfall gradient in northern Australia. Agric. For. Meteorol..

[36] Atkin O.K., Bloomfield K.J., Reich P.B. (2015). Global variability in leaf respiration in relation to climate, plant functional types and leaf traits. New Phytol..

[37] Maire V., Wright I.J., Prentice I.C. (2015). Global effects of soil and climate on leaf photosynthetic traits and rates. Glob. Ecol. Biogeogr..

[38] Smith N.G., Dukes J.S. (2017). LCE: Leaf carbon exchange dataset for tropical, temperate, and boreal species of North and Central America. Ecology.

[39] Dong N., Prentice I.C., Evans B.J. (2017). Leaf nitrogen from first principles: Field evidence for adaptive variation with climate. Biogeosciences.

[40] Wang H., Harrison S.P., Prentice I.C. (2018). The China plant trait database: Towards a comprehensive regional compilation of functional traits for land plants. Ecology.

[41] Bloomfield K.J., Cernusak L.A., Eamus D. (2018). A continental-scale assessment of variability in leaf traits: Within species, across sites and between seasons. Funct. Ecol..

[42] Xu H.Y., Wang H., Prentice I.C. (2021). Predictability of leaf traits with climate and elevation: A case study in Gongga Mountain, China. Tree Physiol..

[43] Harris I., Jones P.D., Osborn T.J. (2014). Updated high resolution grids of monthly climatic observations – the CRU TS3.10 dataset. Int. J. Climatol..

[44] Weedon G.P., Balsamo G., Bellouin N. (2014). The WFDEI meteorological forcing data set: WATCH forcing data methodology applied to ERA-interim reanalysis data. Water Resour. Res..

[45] Davis T.W., Prentice I.C., Stocker B.D. (2017). Simple process-led algorithms for simulating habitats (SPLASH v.1.0): Robust indices of radiation, evapotranspiration and plant-available moisture. Geosci. Model Dev..

[46] Quebbeman J.A., Ramirez J.A. (2016). Optimal allocation of leaf-level nitrogen: Implications for covariation of *V*_cmax_ and *J*_max_ and photosynthetic downregulation. J. Geophys. Res.-Biogeosci..

[47] Goldberg D.E. (1989).

[48] S. Hamblin, On the practical usage of genetic algorithms in ecology and evolution, 4 (2013) 184-194.

[49] Dormann C.F., Elith J., Bacher S. (2013). Collinearity: A review of methods to deal with it and a simulation study evaluating their performance. Ecography.

[50] Doetterl S., Stevens A., Six J. (2015). Soil carbon storage controlled by interactions between geochemistry and climate. Nat. Geosci..

[51] Calcagno V., de Mazancourt C. (2010). glmulti: An R package for easy automated model selection with (generalized) linear models. J. Stat. Softw..

[52] Terrer C., Vicca S., Hungate B.A. (2016). Mycorrhizal association as a primary control of the CO_2_ fertilization effect. Science.

[53] Breheny P., Burchett W W. (2017). Visualization of regression models using visreg. R. J..

[54] Sobol' I.M., Asotsky D., Kreinin A. (2011). Construction and comparison of high-dimensional Sobol' generators. Wilmott.

[55] Saltelli A., Annoni P., Azzini I I. (2010). Variance based sensitivity analysis of model output. Design and estimator for the total sensitivity index. Comput. Phys. Commun..

[56] Yamori W., Hikosaka K., Way D.A. (2014). Temperature response of photosynthesis in C_3_, C_4_, and CAM plants: Temperature acclimation and temperature adaptation. Photosynth. Res..

[57] Galmes J., Kapralov M.V., Copolovici L.O. (2015). Temperature responses of the Rubisco maximum carboxylase activity across domains of life: Phylogenetic signals, trade-offs, and importance for carbon gain. Photosynth. Res..

[58] Kumarathunge D.P., Medlyn B.E., Drake J.E. (2019). Acclimation and adaptation components of the temperature dependence of plant photosynthesis at the global scale. New Phytol..

[59] Wu J., Rogers A., Albert L.P. (2019). Leaf reflectance spectroscopy captures variation in carboxylation capacity across species, canopy environment and leaf age in lowland moist tropical forests. New Phytol..

[60] Yan Z.B., Sardans J., Peñuelas J. (2023). Global patterns and drivers of leaf photosynthetic capacity: The relative importance of environmental factors and evolutionary history. Glob. Ecol. Biogeogr..

[61] Firn J., McGree J.M., Harvey E. (2019). Leaf nutrients, not specific leaf area, are consistent indicators of elevated nutrient inputs. Nat. Ecol. Evol..

[62] Luo X.Z., Keenan T.F., Chen J.M. (2021). Global variation in the fraction of leaf nitrogen allocated to photosynthesis. Nat. Commun..

[63] Evans J.R., Seemann J.R. (1989). The allocation of protein nitrogen in the photosynthetic apparatus: Costs, consequences, and control. Photosynthesis.

[64] Fisher J.B., Sitch S., Malhi Y. (2010). Carbon cost of plant nitrogen acquisition: A mechanistic, globally applicable model of plant nitrogen uptake, retranslocation, and fixation. Glob. Biogeochem. Cycle.

[65] Atkin O.K., Turnbull M.H., Zaragoza-Castells J. (2013). Light inhibition of leaf respiration as soil fertility declines along a post-glacial chronosequence in New Zealand: An analysis using the Kok method. Plant Soil.

[66] Lambers H., Oliveira R.S. (2019).

[67] Liu X.J., Zhang Y., Han W.X. (2013). Enhanced nitrogen deposition over China. Nature.

[68] Peñuelas J J., Janssens I.A., Ciais P. (2020). Anthropogenic global shifts in biospheric N and P concentrations and ratios and their impacts on biodiversity, ecosystem productivity, food security, and human health. Glob. Change Biol..

