# Supplementary material for: Global photosynthetic capacity jointly determined by enzyme kinetics and eco-evo-environmental drivers
Source: Fundam Res. 2024 Feb 6;5(5):2062–72. doi: 10.1016/j.fmre.2023.12.011 (PMC12848234; doi:10.1016/j.fmre.2023.12.011)
Supplement: Supplementary file 1 [file mmc1.docx]

**Supporting Information**

**Title:** Global photosynthetic capacity jointly determined by enzyme kinetics and eco-evo-environmental drivers

**Running head:** Drivers of global photosynthetic capacity

**Author List:** Zhengbing Yan^1,2^, Matteo Detto^3^, Zhengfei Guo^2^, Nicholas G. Smith^4^, Han Wang^5,6^, Loren P. Albert^7^, Xiangtao Xu^8^, Ziyu Lin^2^, Shuwen Liu^2^, Yingyi Zhao^2^, Shuli Chen^9^, Timothy C. Bonebrake^2^, Jin Wu^2,10,11*^

**Author Affiliations:**

(1) State Key Laboratory of Vegetation and Environmental Change, Institute of Botany, Chinese Academy of Sciences, Xiangshan, Beijing, China

(2) School of Biological Sciences, The University of Hong Kong, Hong Kong Special Administrative Region, China

(3) Department of Ecology and Evolutionary Biology, Princeton University, Princeton, NJ 08544, USA

(4) Department of Biological Sciences, Texas Tech University, Lubbock, TX USA

(5) Ministry of Education Key Laboratory for Earth System Modelling, Department of Earth System Science, Tsinghua University, Beijing, China

(6) Joint Centre for Global Change Studies, Tsinghua University, Beijing, China

(7) Department of Biology, West Virginia University, Morgantown, West Virginia 26506 USA

(8) Department of Ecology and Evolutionary Biology, Cornell University, Ithaca, NY, 14853

(9) Department of Ecology and Evolutionary Biology, University of Arizona, Tucson, AZ 85721, USA

(10) Institute for Climate and Carbon Neutrality, The University of Hong Kong, Hong Kong, China

(11) State Key Laboratory of Agrobiotechnology, The Chinese University of Hong Kong, Shatin, Hong Kong, China

*** Corresponding Author:** Jin Wu

School of Biological Sciences, The University of Hong Kong, Pokfulam Road, Hong Kong Special Administrative Region, China (email: jinwu@hku.hk; phone: +852 2299-0655)


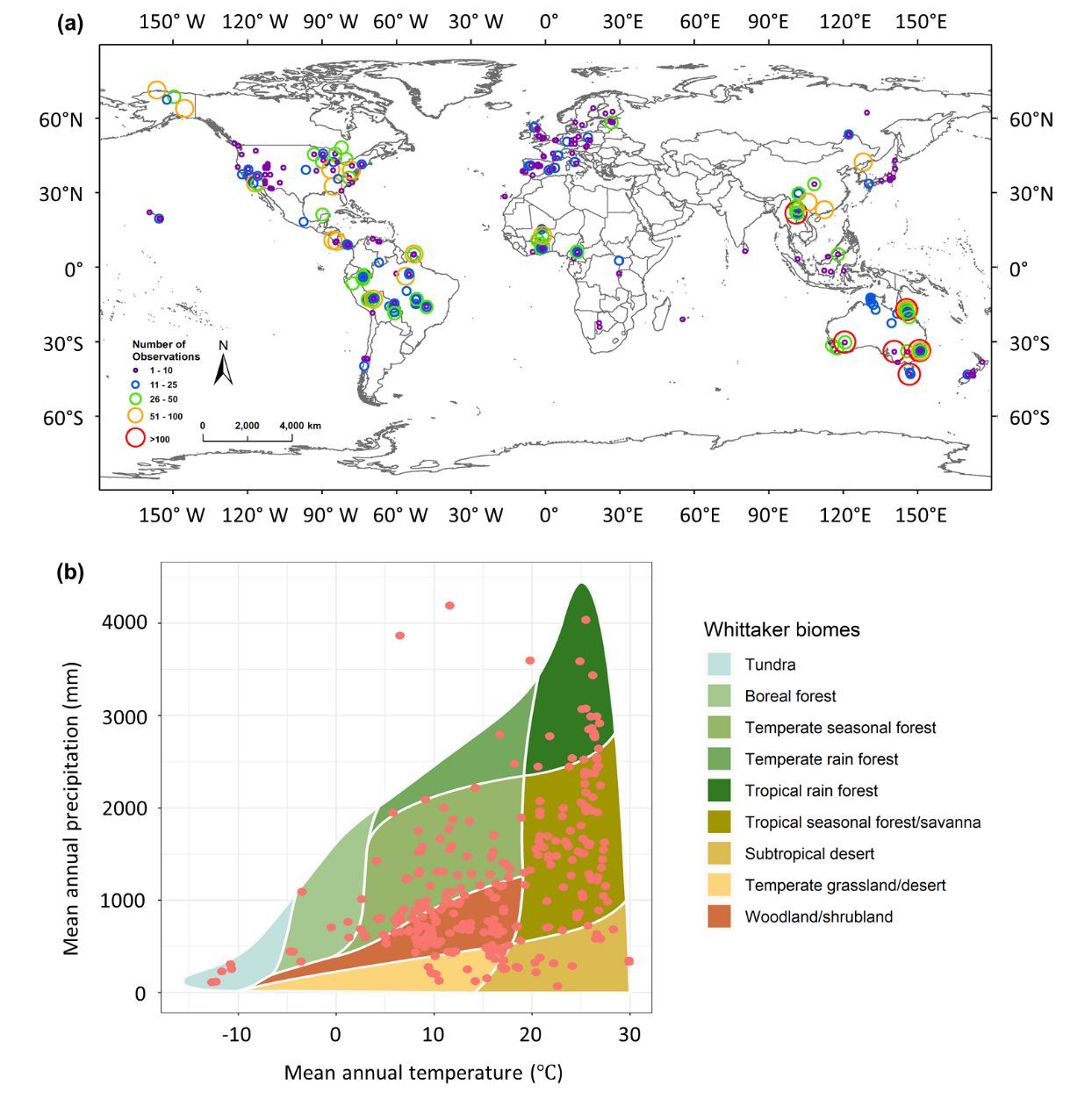
**Fig. S1** Site distribution of the newly compiled field-measured *V*_c,max_ dataset (*n*=7339 records from 428 sites) for C_3_ plants worldwide. (a) Location of each sampling site in a background of world map. The points with different color and size indicate the sites with different numbers of observations. (b) Location of each sampling site superimposed upon classic Whittaker Biome Classification by climate.


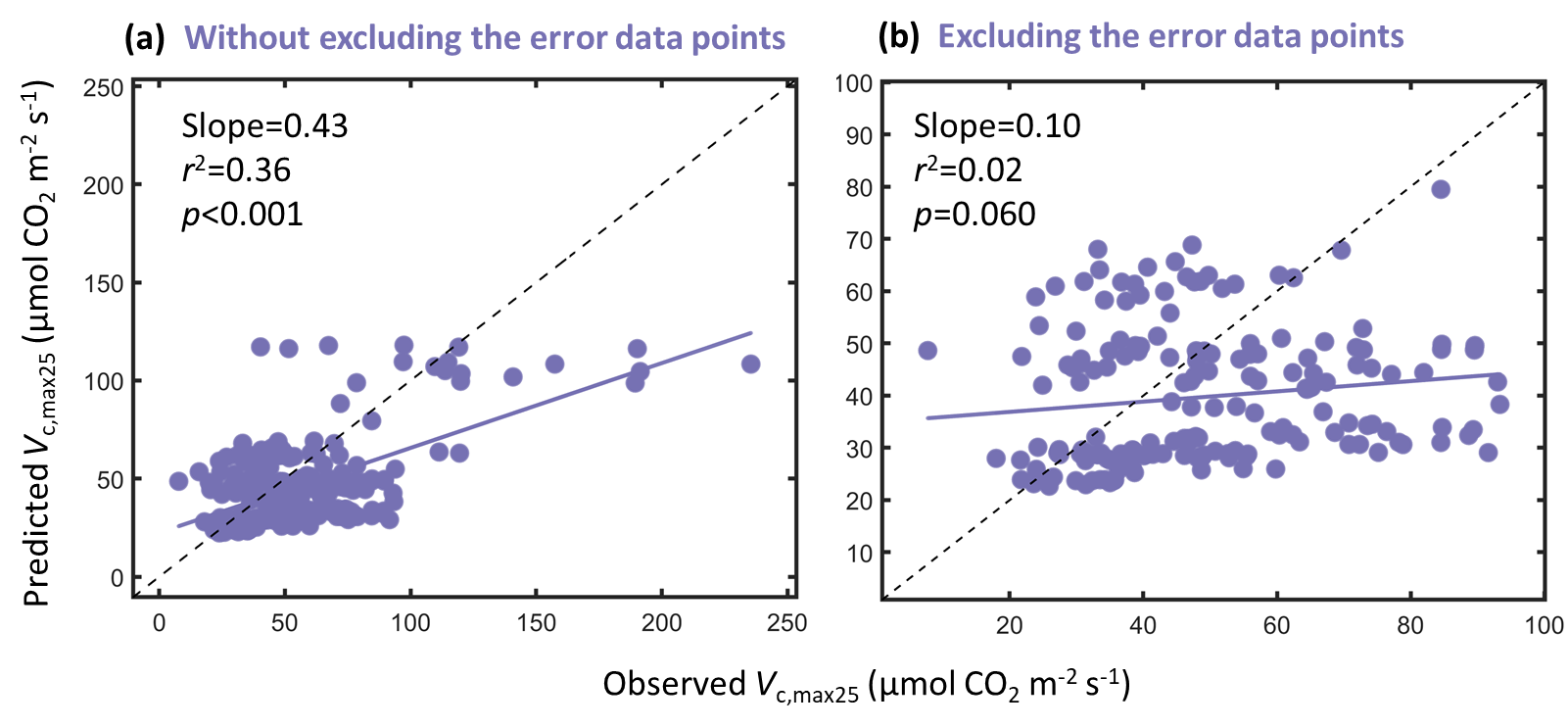
**Fig. S2** The optimality-constant model performance in predicting *V*_c,max25_ using the Peng *et al.* (2021) dataset [1]. In panel (a), we conducted the model estimation by use of the data from Peng et al. (2021) involving those records with the growing-season temperature as the proxy of leaf measurement temperature was used for the optimality-constant model estimation [1]; in panel (b), we removed those records with the growing-season temperature as the proxy of leaf measurement temperature, and re-estimated the optimality-constant model performance with the revised Peng et al. (2021) dataset.


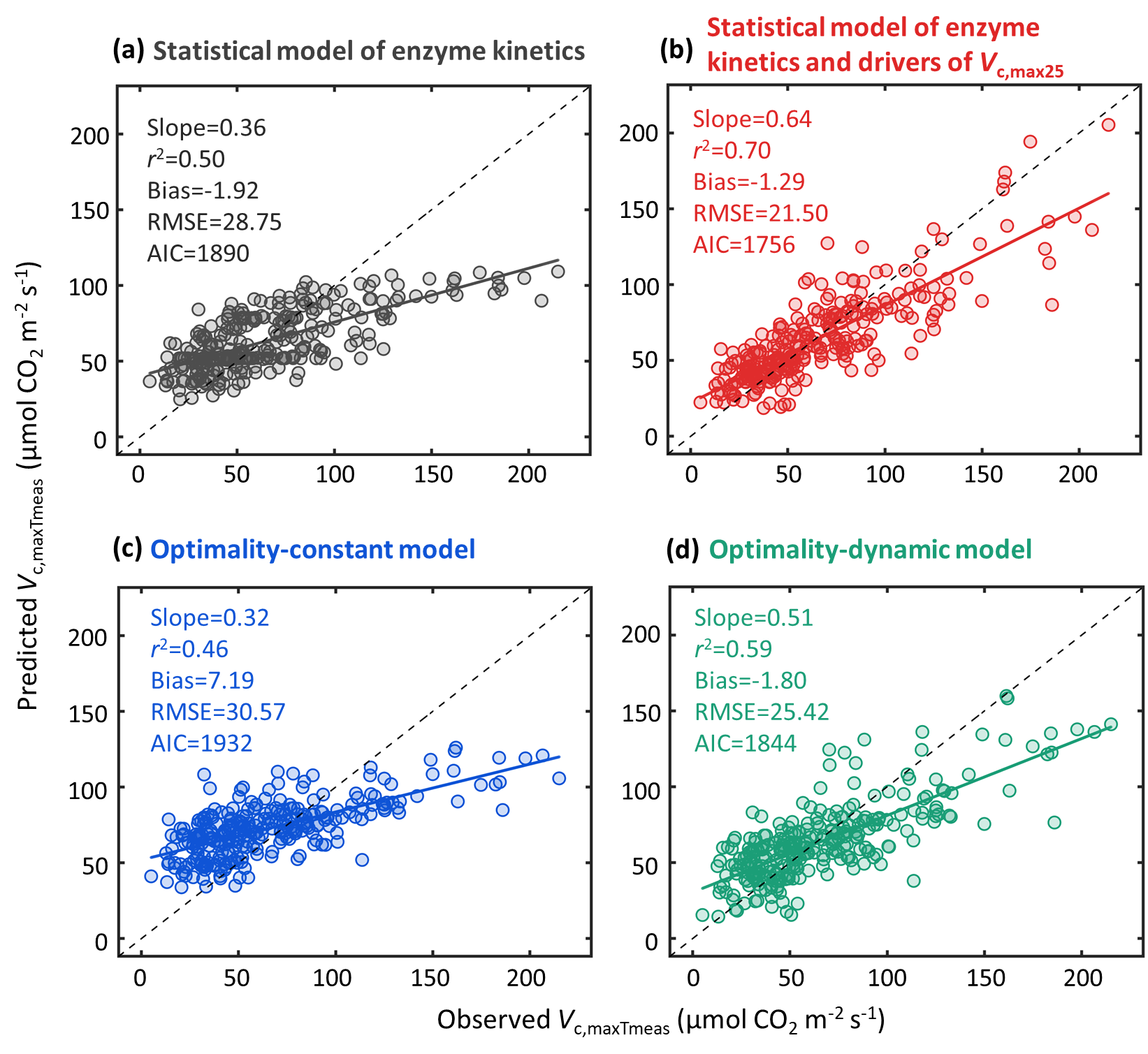
**Fig.** **S3** The statistical model considering both enzyme kinetics and eco-evo-environmental drivers of *V*_c,max25_ outperforms the optimality model in predicting global *V*_c,maxTmeas_ (i.e. *V*_c,max_ derived at its measurement temperature) variability. The statistical modelling approach is analyzed at two levels: global *V*_c,maxTmeas_ variability is described (a) using the temperature-associated enzyme kinetics together with a globally averaged *V*_c,max25_, or (b) by adding temperature-associated enzyme kineticsto site-specific *V*_c,max25_ derived from its empirical relationship with both environmental variables and leaf traits. The optimality model is used for global *V*_c,maxTmeas_ prediction with (c) a default globally constant *c* (i.e. the total unit carbon cost of building and maintaining the photosynthetic machinery), or the optimality-constant model, or (d) a site-specific dynamic *c* constrained by edaphic variables, or the optimality-dynamic model.


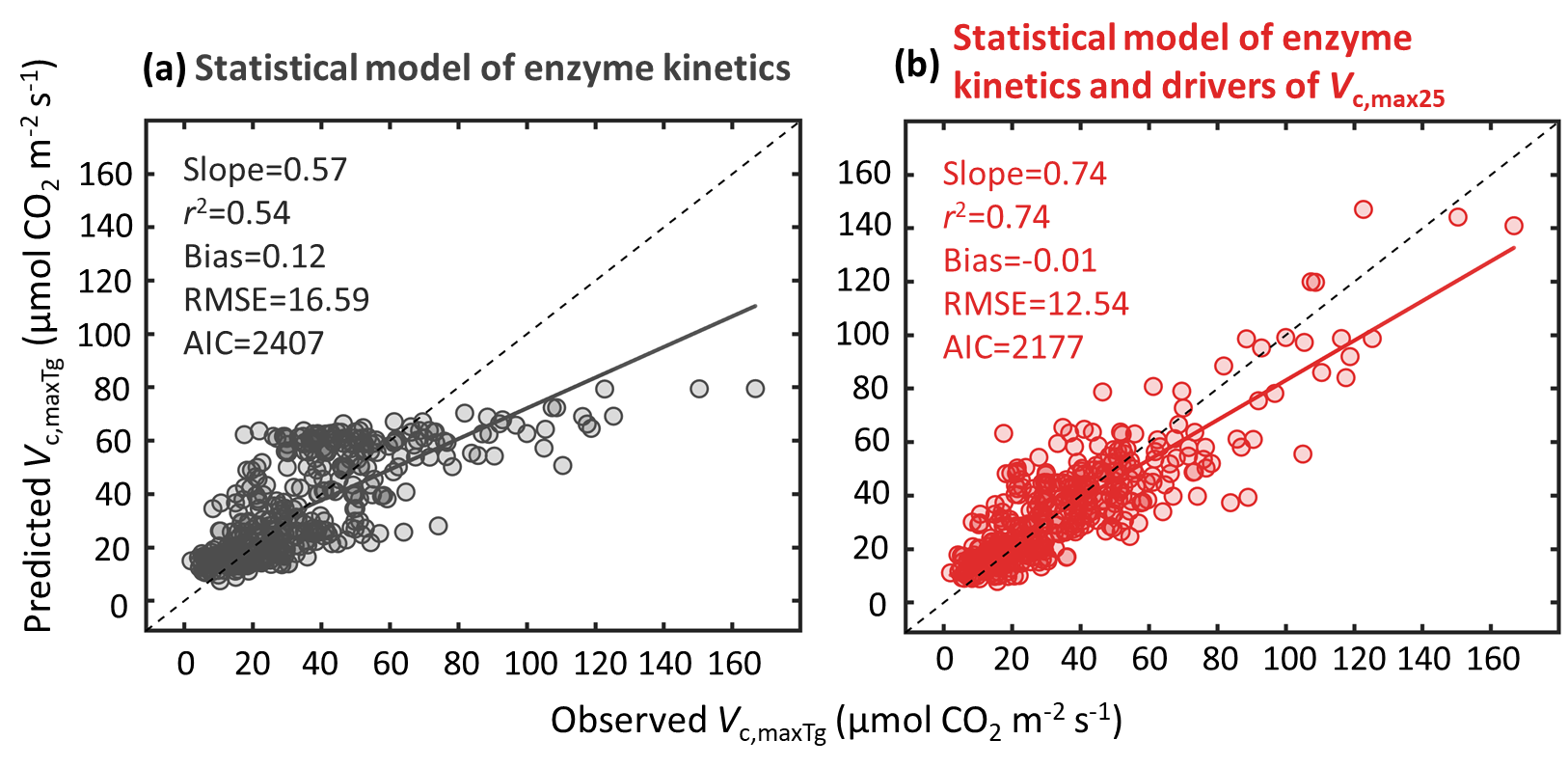
**Fig.** **S4** The relative importance of enzyme kinetics and eco-evo-environmental drivers of *V*_c,max25_ in controlling global *V*_c,maxTg_ variability for the entire dataset. In this analysis, the entire dataset with concurrent measurements of both aboveground environmental variables (i.e. mean growing-season temperature (*T*_g_), vapor pressure deficit (VPD), incoming photosynthetically active radiation (PAR), precipitation, atmosphere CO_2_ concentration (*C*_a_) and elevation) and *V*_c,max_ was used. The statistical modelling approach is analyzed at two levels: global *V*_c,maxTg_ variability is described (a) using the temperature-associated enzyme kinetics together with a globally averaged *V*_c,max25_, or (b) by adding temperature-associated enzyme kineticsto site-specific *V*_c,max25_ derived from its empirical relationship with the aboveground environmental variables.


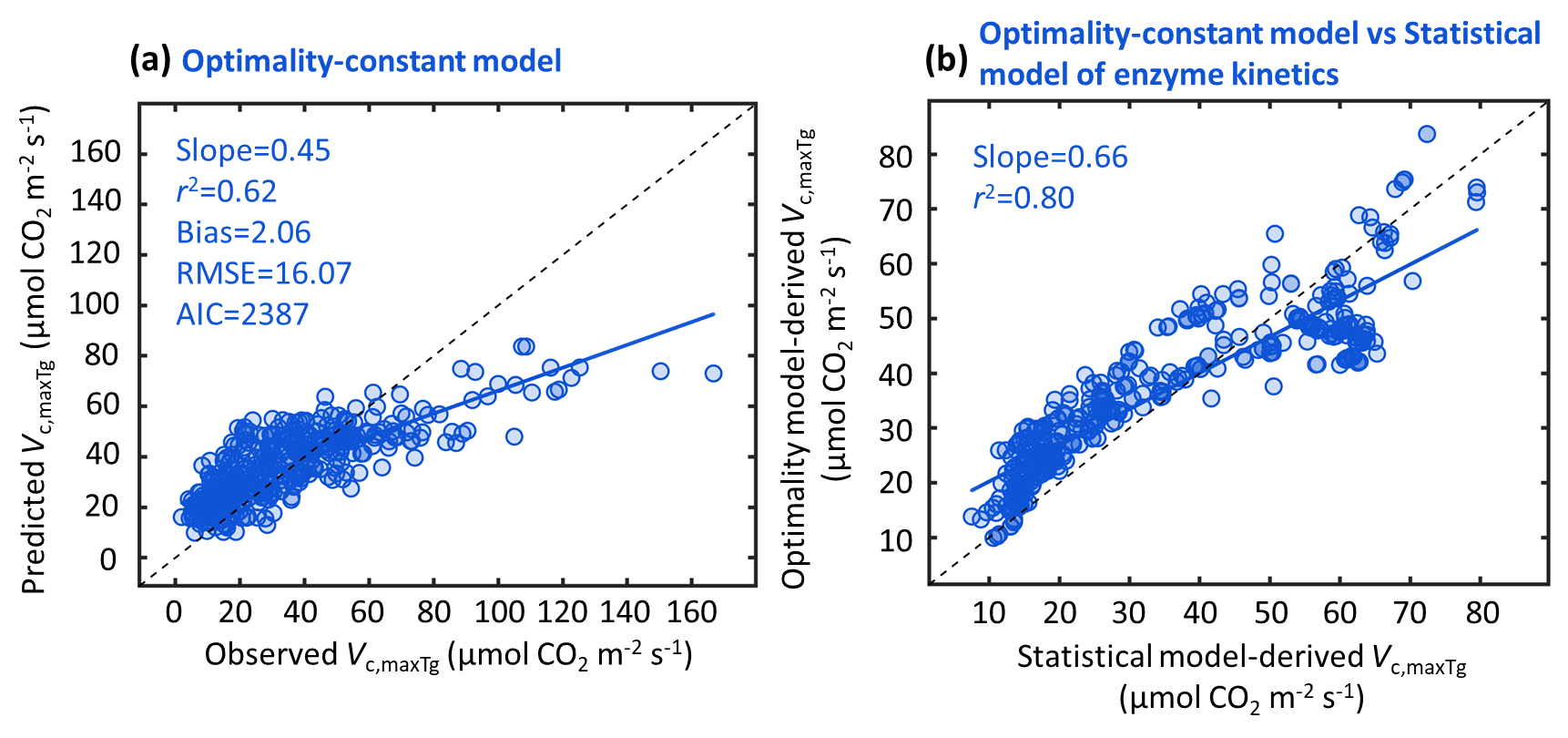
**Fig.** **S5** The performance of optimality-constant model in predicting global *V*_c,maxTg_ variability for the entire dataset. The optimality model is used for global *V*_c,maxTg_ prediction with a default globally constant *c* (i.e. the total unit carbon cost of building and maintaining the photosynthetic machinery), or the optimality-constant model (a), which displays high correlation as the statistical model of enzyme kinetics (b). The statistical model of enzyme kinetics here is the same as Fig. S3a.


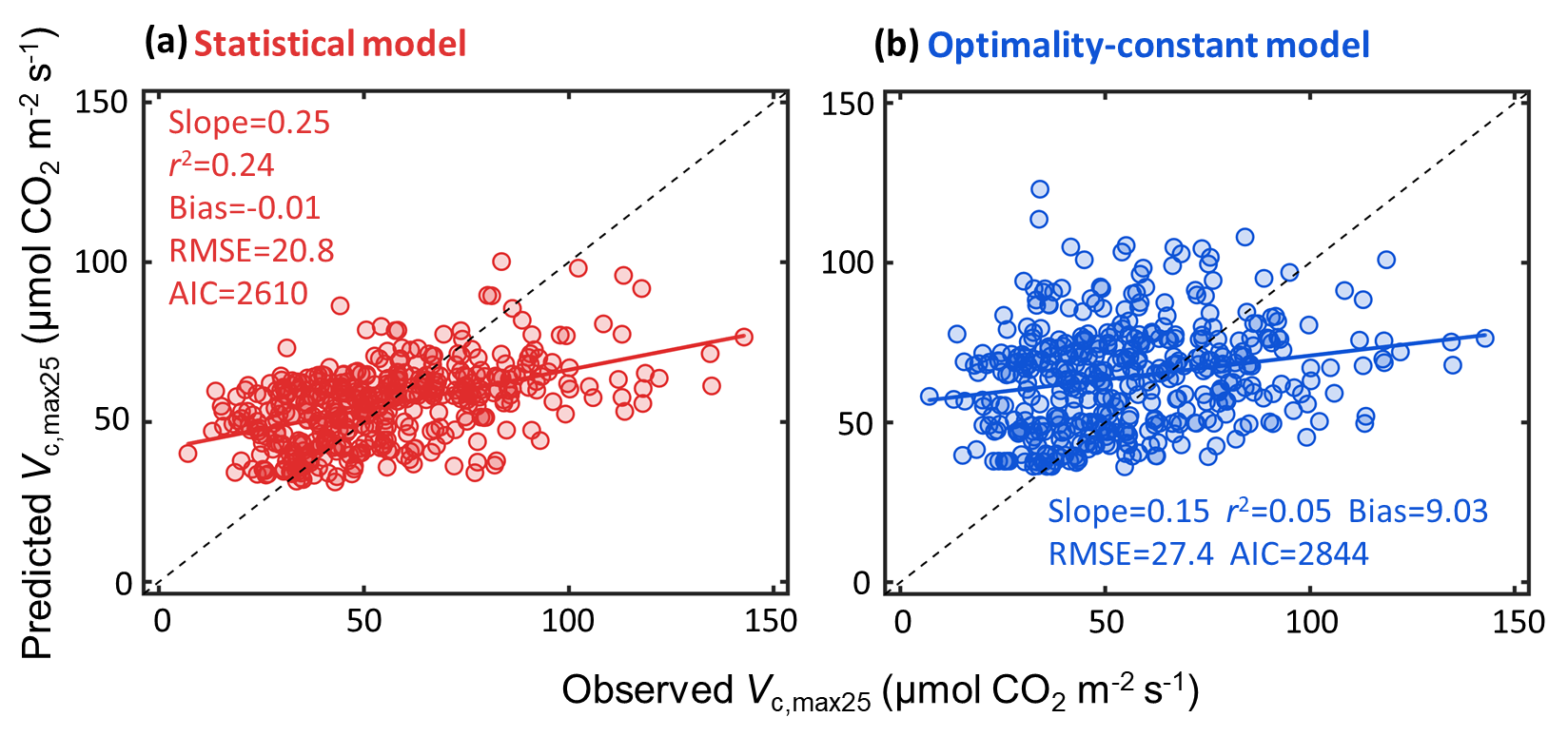
**Fig.** **S6** The statistical model outperforms the optimality-constant model in predicting *V*_c,max25_ for the entire dataset. The comparison of field-derived *V*_c,max25_ with (a) the statistical model derived *V*_c,max25_ that relies on its multiple linear regression relationship with the aboveground environmental variables, and (b) the *V*_c,max25_ derived from the optimality-constant model.


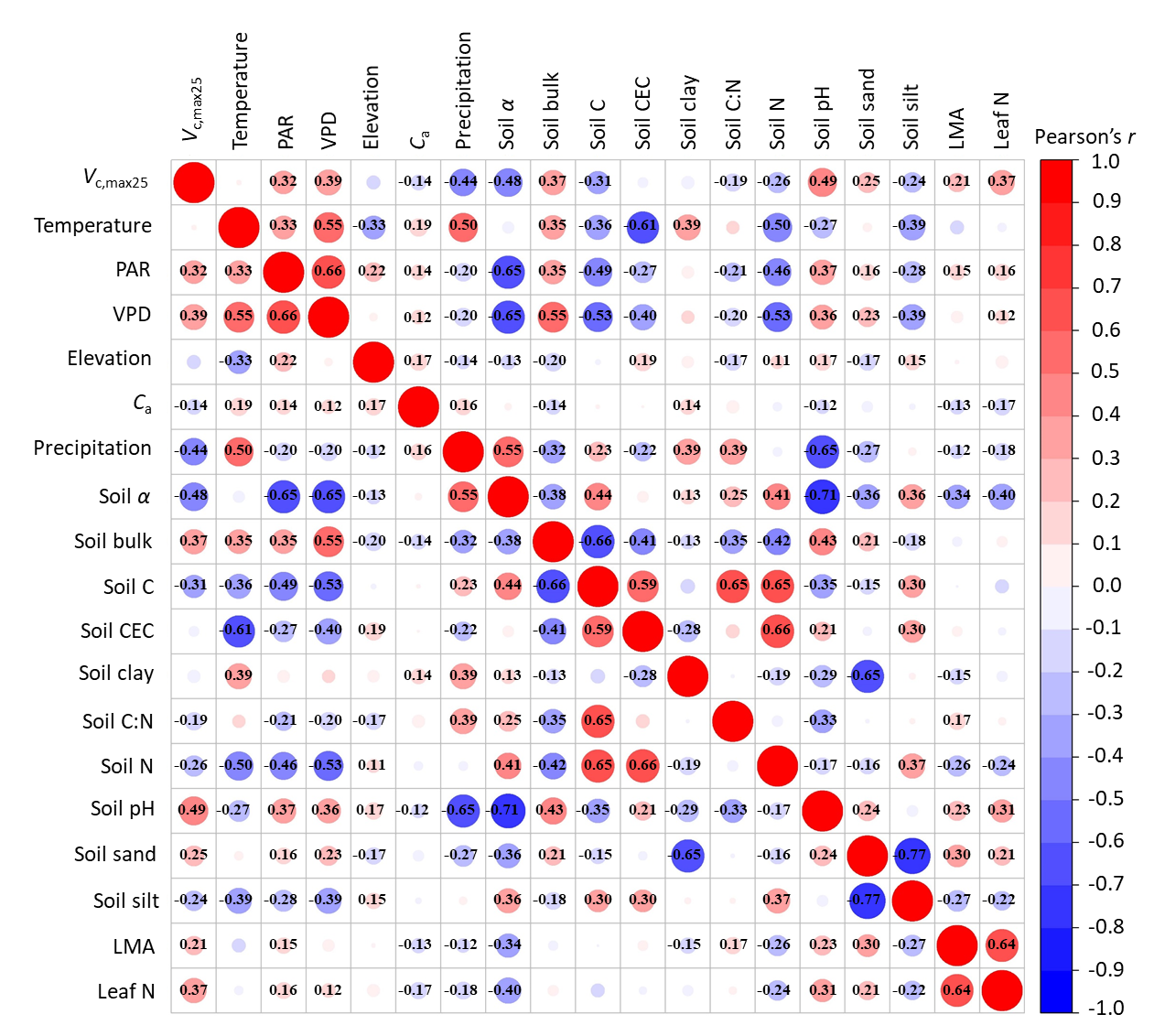
**Fig.** **S7** Correlations among all examined environmental variables, leaf traits and *V*_c,max25_. The circles with different size and colour denote the strength and sign of the correlation. The significant (*p*<0.05) correlation coefficients among them are presented here. The detailed descriptions about the aboveground environmental variables, edaphic variables and leaf traits are shown in the Methods section.

**
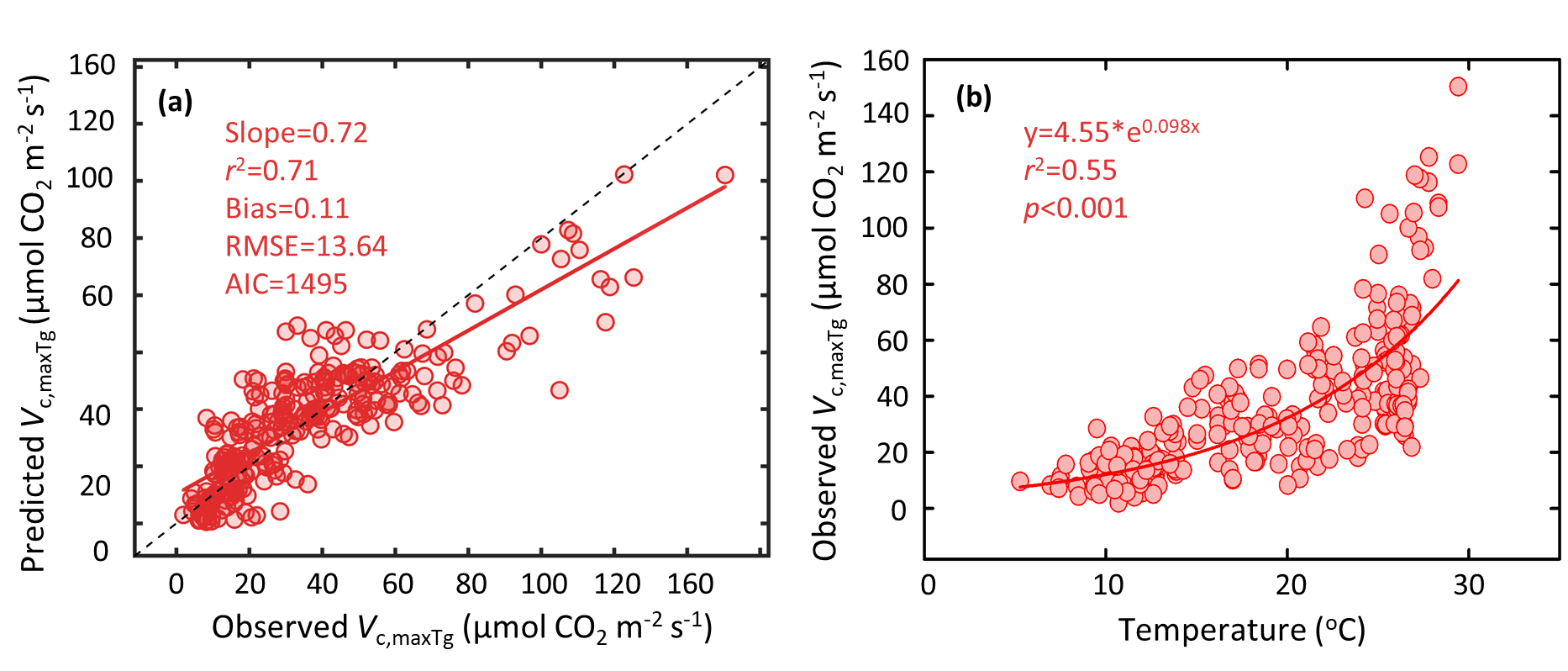
Fig. S8** A comparison of field-derived *V*_c,maxTg_ and the statistical model-derived *V*_c,maxTg_ based on its multiple linear regression relationship with both environmental variables and leaf traits (a), as well as the ordinary least-square regression plot of observed *V*_c,maxTg_ against the predictor of growing-season mean temperature (b). Five statistical metrics used to assess model performance: 1) *r*^2^-the square of correlation coefficient, 2) Bias-the residual bias, 3) RMSE-the root mean square of error, 4) AIC-Akaike Information Criterion, and 5) *p*-value-the square of correlation coefficient and significant level, respectively. Lines are fitted using ordinary least-square regressions.


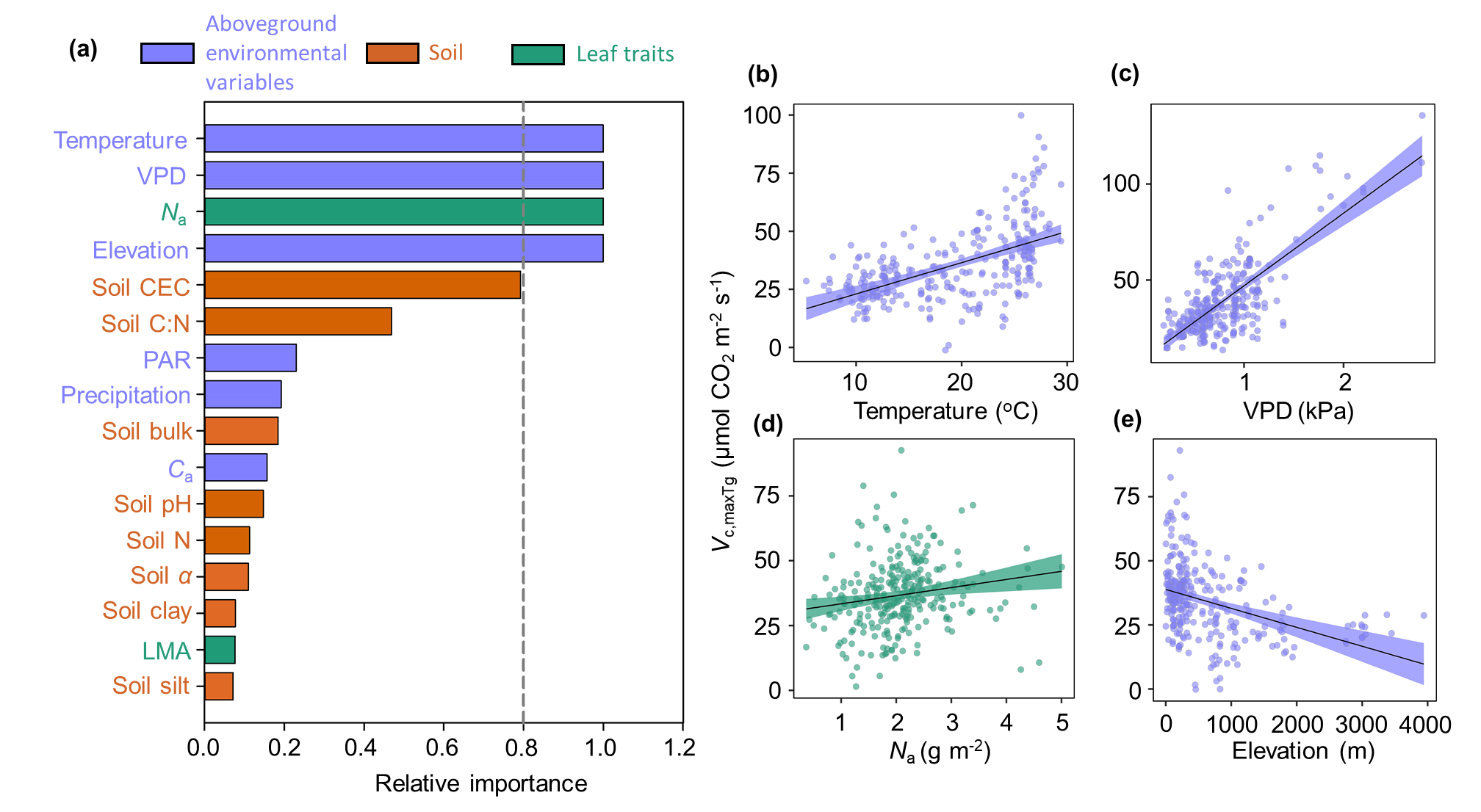
**Fig. S9** Relative importance of environmental variables and leaf traits in predicting global *V*_c,maxTg_ variability. (a) The relative importance of each variable based on the sum of the Akaike weights derived from a model selection using corrected AIC; (b-e) partial regression plots of *V*_c,max25_ with the predictor of growing-season mean temperature, vapor pressure deficit (VPD), area-based leaf nitrogen content (*N*_a_), and elevation, respectively. The cutoff (dashed line) of panel (a) is set at 0.8 for identifying the most important predictor variables; the shade area in (b-e) represent 95% confidential intervals around the predicted relationships.

**
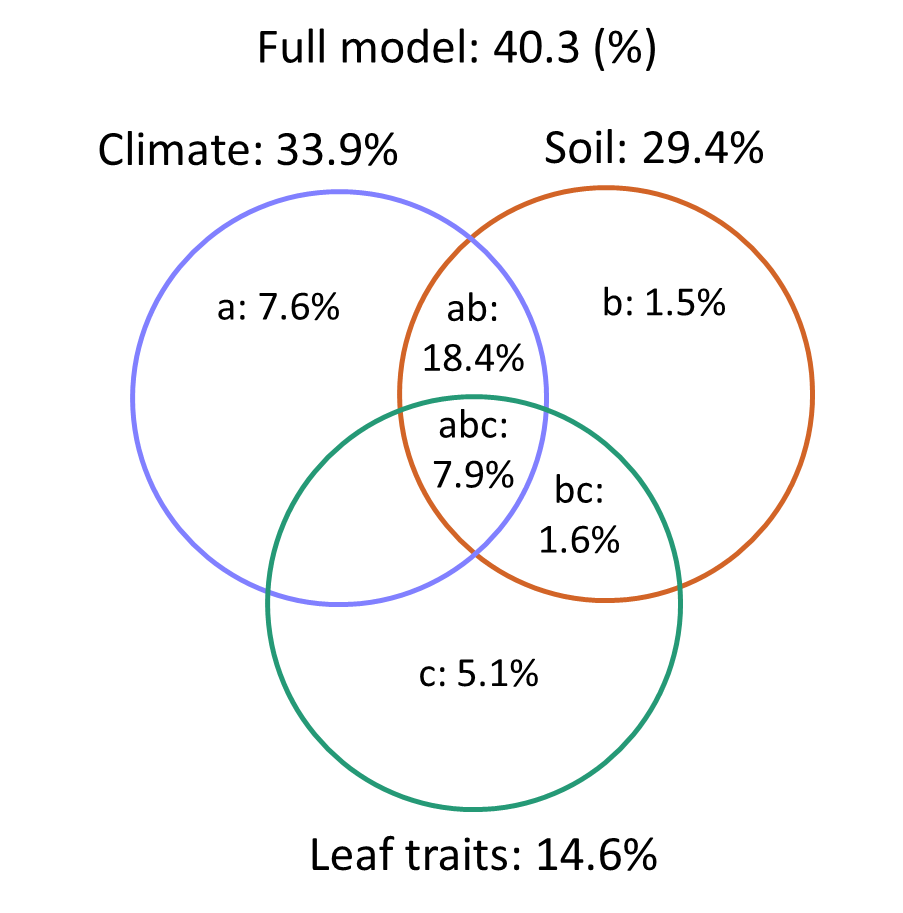
Fig. S10** Variation partitioning (*r*^2^) of climate variables, soil properties and leaf traits in accounting for the variations in global *V*_c,max25_ variability. The symbols a, b and c represent the independent effects of climate, soil, and leaf traits, respectively; ab denotes the interactive effect of climate and soil; bc signifies the interactive effect of soil and leaf traits; and abc indicates the interactive effect of climate, soil and leaf traits. Note: In the full model, any explained variation less than 0 is disregarded.

**Table S1** Statistical models of environmental variables and leaf traits in predicting global-scale *V*_c,max25_ variability. The multiple linear regression analysis is used to quantify the separate and joint effects of environmental factors and leaf traits on *V*_c,max25_ variability. Four statistical metrics for assessing multiple linear model performance include 1) *r*^2^–the square of correlation coefficient, 2) Bias–the residual bias, 3) RMSE–the root mean square of error, and 4) AIC–Akaike Information Criterion.

| Scenarios | Response | Predictor | Intercept | Slope | *r*^2^ | *p*-value | Bias | RMSE | AIC |
| --- | --- | --- | --- | --- | --- | --- | --- | --- | --- |
| Model with the predictors of environmental variables alone | Observed *V*_c,max25_ | Predicted *V*_c,max25_ | 5.66 | 0.89 | 0.31 | <0.001 | -0.153 | 18.13 | 1656 |
| Model with the predictors of leaf traits alone | Observed *V*_c,max25_ | Predicted *V*_c,max25_ | 4.75 | 0.91 | 0.11 | <0.001 | -0.001 | 20.57 | 1703 |
| Model with the predictors of both environmental variables and leaf traits | Observed *V*_c,max25_ | Predicted *V*_c,max25_ | 5.17 | 0.90 | 0.36 | <0.001 | -0.124 | 17.48 | 1640 |

**Table S2** The optimality models in predicting global *V*_c,maxTg_ variability with *c* (i.e. the total unit carbon cost of building and maintaining the photosynthetic machinery) parameterized under the following four scenarios: (1) a default globally constant *c* at 0.053 as the current optimality model [2], and a site-specific dynamic *c* respectively constrained by (2) the edaphic variables alone, (3) all environmental variables and leaf traits, and (4) the five most important variables (i.e. *N*_a_, VPD, soil pH, elevation, and precipitation) identified from the statistical modelling (Fig. 4). Four statistical metrics for assessing model performance include 1) *r*^2^–the square of correlation coefficient, 2) Bias–the residual bias, 3) RMSE–the root mean square of error, and 4) AIC–Akaike Information Criterion.

| Scenarios | Response | Predictor | Intercept | Slope | *r*^2^ | *p*-value | Bias | RMSE | AIC |
| --- | --- | --- | --- | --- | --- | --- | --- | --- | --- |
| Default constant *c* | Observed *V*_c,maxTg_ | Predicted *V*_c,maxTg_ | -21.51 | 1.50 | 0.67 | <0.001 | 2.50 | 16.38 | 1581 |
| *c* constrained by edaphic variables | Observed *V*_c,maxTg_ | Predicted *V*_c,maxTg_ | -6.45 | 1.25 | 0.74 | <0.001 | -2.04 | 13.80 | 1501 |
| *c* constrained by all environmental variables and leaf traits | Observed *V*_c,maxTg_ | Predicted *V*_c,maxTg_ | -4.80 | 1.18 | 0.73 | <0.001 | -1.43 | 13.24 | 1472 |
| *c* constrained by the five important variables that drive global *V*_c,max25_ variability | Observed *V*_c,maxTg_ | Predicted *V*_c,maxTg_ | -8.98 | 1.23 | 0.74 | <0.001 | 0.59 | 13.19 | 1462 |

**Table S3** The optimality models in predicting global *V*_c,max25_ variability with *c* (i.e. the total unit carbon cost of building and maintaining the photosynthetic machinery) parameterized under the following four scenarios: (1) a default globally constant *c* at 0.053 as the current optimality model [2], and a site-specific dynamic *c* respectively constrained by (2) the edaphic variables alone, (3) all environmental variables and leaf traits, and (4) the five most important variables (i.e. *N*_a_, VPD, soil pH, elevation, and precipitation) identified from the statistical modelling (Fig. 4). Four statistical metrics for assessing model performance include 1) *r*^2^–the square of correlation coefficient, 2) Bias–the residual bias, 3) RMSE–the root mean square of error, and 4) AIC–Akaike Information Criterion.

| Scenarios | Response | Predictor | Intercept | Slope | *r*^2^ | *p*-value | Bias | RMSE | AIC |
| --- | --- | --- | --- | --- | --- | --- | --- | --- | --- |
| Default constant *c* | Observed *V*_c,max25_ | Predicted *V*_c,max25_ | 38.32 | 0.22 | 0.03 | <0.001 | 10.29 | 26.95 | 1861 |
| *c* constrained by edaphic variables | Observed *V*_c,max25_ | Predicted *V*_c,max25_ | 23.74 | 0.54 | 0.19 | <0.001 | 0.42 | 21.23 | 1743 |
| *c* constrained by all environmental variables and leaf traits | Observed *V*_c,max25_ | Predicted *V*_c,max25_ | 15.11 | 0.71 | 0.22 | <0.001 | -0.30 | 19.57 | 1691 |
| *c* constrained by the five important variables that drive global *V*_c,max25_ variability | Observed *V*_c,max25_ | Predicted *V*_c,max25_ | 20.96 | 0.55 | 0.19 | <0.001 | 4.83 | 21.57 | 1738 |

**Table S4** Details of all models with the fitted equations under different scenarios. In this table, *f*(25, *T*_g_) is the temperature response function (see details in Equations (2-3) in Materials and Methods); and *f*(*T*_g_, VPD, PAR, *C*_a_, *z*, *c*) represents the optimality-dynamic model (see details in “The optimality theory” sections in Materials and Methods). The predictors for these models include climate variables, soil variables and leaf traits. Specifically, the climate variables include the mean growing-season temperature (*T*_g_, ℃), vapour pressure deficit (VPD, kPa), incoming photosynthetically active radiation (PAR, μmol m^-2^ s^-1^), precipitation (*P*, mm), atmosphere CO_2_ concentration (*C*_a_, umol mol^-1^) and elevation (*z*, m); soil variables include the pH (unitless), nitrogen (N) content (g kg^-1^), C:N ratio (unitless), the ratio of actual evapotranspiration to equilibrium evapotranspiration (*α*, unitless), cation exchange capacity (CEC, cmol_c_ kg^-1^), silt content (Silt, %), clay content (Clay, %) and bulk density (BD, kg dm^-3^); and leaf traits include leaf mass per area (LMA, g m^-2^) and leaf N content per unit leaf area (*N*_a_, g m^-2^). The cost factor *c* is defined as the total unit carbon cost of building and maintaining the two components of photosynthetic machinery (see details in Equation 6 in Materials and Methods).

| Figure | Scenarios | Response | Predictors | Equations |
| --- | --- | --- | --- | --- |
| Fig. 1a | Statistical model of enzyme kinetics | *V*_c,maxTg_ | *T*_g_ | *V*_c,maxTg_=51.94×*f*(25,*T*_g_) |
| Fig. 1b | Statistical model of enzyme kinetics and drivers of *V*_c,max25_ | *V*_c,maxTg_ | *T*_g_, VPD, PAR, *P*, *C*_a_, *z*, pH, N, C:N, *α*, CEC, Silt, Clay, BD, LMA, *N*_a_ | *V*_c,maxTg_=(a_0_+a_1_×*T*_g_+a_2_×VPD+a_3_×PAR+a_4_×*P*+a_5_×*C*_a_+a_6_×*z*+a_7_×pH+a_8_×N+a_9_×C:N+a_10_×*α*+a_11_×CEC+a_12_×Silt+a_13_×Clay+a_14_×BD+a_15_×LMA+a_16_×*N*_a_)×*f*(25,*T*_g_) |
| Fig. 3a | Statistical model of enzyme kinetics and drivers of *V*_c,max25_ | *V*_c,max25_ | *T*_g_, VPD, PAR, *P*, *C*_a_, *z*, pH, N, C:N, *α*, CEC, Silt, Clay, BD, LMA, *N*_a_ | *V*_c,max25_=a_0_+a_1_×*T*_g_+a_2_×VPD+a_3_×PAR+a_4_×*P*+a_5_×*C*_a_+a_6_×*z*+a_7_×pH+a_8_×N+a_9_×C:N+a_10_×*α*+a_11_×CEC+a_12_×Silt+a_13_×Clay+a_14_×BD+a_15_×LMA+a_16_×*N*_a_ |
| Fig. 5 | Optimality-dynamic model with the cost factor *c* constrained by edaphic variables | *V*_c,maxTg/_ *V*_c,max25_ | *T*_g_, VPD, PAR, *C*_a_, *z*, pH, N, C:N, *α*, CEC, Silt, Clay, BD | *V*_c,maxTg or_ *V*_c,max25_=*f*(*T*_g_, VPD, PAR, *C*_a_, *z*, *c*) with *c*=b_0_+b_1_×pH+b_2_×N+b_3_×C:N+b_4_×*α*+b_5_×CEC+b_6_×Silt+b_7_×Clay+b_8_×BD |

**Table S5** Mean values and 95% confidence intervals (CIs) for the coefficients of the predictors in Table S4. These coefficients are derived from the multiple linear regression models with 5-fold cross-validation and 100 repetitions (see details in Materials and Methods). The coefficients for the predictors are denoted as a_0_, a_1_, a_2_, …, and a_16_ in the statistical model of enzyme kinetics and drivers of *V*_c,max25_, and b_0_, b_1_, b_2_, …, and b_8_ in the optimality-dynamic model.

| Coefficients | Mean (95% CI) | Coefficients | Mean (95% CI) |
| --- | --- | --- | --- |
| a_0_ | 52.10 (51.04, 53,16) | b_0_ | 0.116 (0.060, 0.172) |
| a_1_ | -3.33 (-6.19, -0.48) | b_1_ | 0.005 (-0.061, 0.070) |
| a_2_ | 5.99 (3.86, 8.11) | b_2_ | 0.069 (0.006, 0.131) |
| a_3_ | 3.15 (1.37, 4.92) | b_3_ | -0.009 (-0.063, 0.045) |
| a_4_ | -4.78 (-6.94, -2.63) | b_4_ | 0.019 (-0.025, 0.064) |
| a_5_ | -0.81 (-1.92, 0.29) | b_5_ | -0.017 (-0.057, 0.024) |
| a_6_ | -5.60 (-7.08, -4.13) | b_6_ | 0.057 (0.014, 0.100) |
| a_7_ | 4.56 (2.41, 6.70) | b_7_ | -0.022 (-0.065, 0.022) |
| a_8_ | -1.87 (-4.07, 0.32) | b_8_ | -0.131 (-0.170, -0.091) |
| a_9_ | -0.35 (-1.37, 0.67) |  |  |
| a_10_ | 3.85 (1.33, 6.38) |  |  |
| a_11_ | 1.77 (-0.51, 4.06) |  |  |
| a_12_ | -2.29 (-3.56, -1.01) |  |  |
| a_13_ | 1.87 (0.39, 3.34) |  |  |
| a_14_ | 0.66 (-1.30, 2.63) |  |  |
| a_15_ | -2.23 (-4.04, -0.43) |  |  |
| a_16_ | 6.84 (5.10, 8.59) |  |  |

**Supporting Method S1** Model formulations of the optimality model of *V*_c,maxTg_

The first optimization takes advantage of photosynthetic coordination hypothesis stating that photosynthetic machinery acclimates to their growing environments in a way that the rate of Rubisco carboxylation-limited photosynthesis (*A*_c_) is at equilibrium with the photosynthetic rate (*A*_j_) subject to the limitation of electron transport rate [3]:

$A_{c}=A_{j}$ (S1)

*A*_c_ and *A*_j_ can be described using the classic biochemical model of photosynthesis [4], with the following two equations:

$A_{c}=V_{c,max}\times\frac{C_{i}-\Gamma^{*}}{C_{i}+K}$ (S2)

$A_{j}=\frac{J}{4}\times\frac{C_{i}-\Gamma^{*}}{C_{i}+2\Gamma^{*}}$ (S3)

Where *C*_i_ can be estimated using the least-cost theory (see details below). *Γ*^*^ (Pa) is CO_2_ compensation point in the absence of mitochondrial respiration, and *K* (Pa) is Michaelis–Menten coefficient of RuBisCO activity. *Γ*^*^ and *K* are both temperature-dependent, and can be derived at a given temperature using the equations and parameters of Bernacchi *et al.* (2001) [4]. *J* is electron transport rate for the regeneration of ribulose-1,5-bisphosphate (μmol m^-2^ s^-1^) and can be described as a non-rectangular hyperbolic function of irradiance [5]:

$\theta J^{2}-\left( \varphi I+J_{max} \right)J+\varphi IJ_{max}=0$ (S4)

Where irradiance *I* is incident photosynthetically active photon flux density (μmol m^−2^ s^−1^); *θ* (unitless; set to 0.85) is the curvature of light response curve; and *φ* is the realized quantum yield of photosynthetic electron transport (mol mol^-1^). *φ* is parameterized following the temperature function by Bernacchi *et al.* (2003) [6].

$\varphi=a_{L}\times b_{L}\times(0.352+0.022T_{g}-0.00034{T_{g}}^{2})$ (S5)

Where $a_{L}$ is leaf absorptance, and $b_{L}$ is the fraction of absorbed light that reaches photosystem II. The product $a_{L}\times b_{L}$ was fitted to the data to ensure the slope closest to 1:1 line with the value of 0.165, following Smith and Keenan (2020) [7].

Equation (S5) can be substituted into equation (S4) to yield

$A_{j}=\frac{m}{4}\frac{\varphi I+J_{max}\pm\sqrt{\left( \varphi I+J_{max} \right)^{2}-4\theta\varphi IJ_{max}}}{2\theta}$ (S6)

from which the smaller root is used to derive *A*_j_, and *m* is $\frac{C_{i}-\Gamma^{*}}{C_{i}+2\Gamma^{*}}$.

The optimal *J*_max_ can be derived based on the first optimization criterion in Equation (6), which can then be expressed as

$\frac{\partial A_{j}}{\partial J_{max}}=c$ (S7)

Taking the derivative of *A*_j_ with respect to *J*_max_ in equation (S6) leads to

$\frac{m}{8\theta}\left( 1-\frac{\varphi I+J_{max}-2\theta\varphi I}{\sqrt{\left( \varphi I+J_{max} \right)^{2}-4\theta\varphi IJ_{max}}} \right)=c$ (S8)

which can be solved for the optimal *J*_max_ as:

$J_{max}=\varphi I\varpi$ (S9)

where

$\varpi=-\left( 1-2\theta\right)+\sqrt{\left( 1-\theta\right)\left( \frac{1}{\frac{4c}{m}\left( 1-\theta\frac{4c}{m} \right)}-4\theta\right)}$ (S10)

Despite *c* could vary considerably across different sites, in the current optimality model as shown in Smith *et al* (2019) [2], *c* is set as a constant across all sites, with a default value of 0.053 obtained by inverting the model under standard conditions (i.e., *T*_g_=25°C, elevation=0 km, VPD=1 kPa, and *C*_a_=360 ppm) and imposing a linear relationship between *J*_max,25_ and *V*_c,max,25_.(i.e. *J*_max,25_ = 2.07 *V*_c,max,25_). In this study we compared solutions obtained with *c* = 0.053, with solutions where *c* is variable across sites and function of environmental factors and leaf traits (see below).

Combining the Equations (S6), (S9) and (S10) gives:

$A_{j}=\frac{\varphi Im\varpi^{*}}{8\theta}$ (S11)

$\varpi^{*}$ can be derived from the following two equations:

$\varpi^{*}=1+\varpi-\sqrt{\left( 1+\varpi\right)^{2}-4\theta\varpi}$ (S12)

Therefore, by combining the Equations (S1), (S2) and (S11), we derived the optimal *V*_c,max_ (termed as *V*_c,max_^*^) below:

${V_{c,max}}^{*}=\varphi I\frac{C_{i}+K}{C_{i}+2\Gamma^{*}}\frac{\varpi^{*}}{8\theta}$ (S13)

Where the optimal *C*_i_ can be estimated using the least-cost theory [8] illustrated below.

Based on the second optimization criterion in Equation (5), the least cost theory can be determined below

$a\frac{\partial(E/A)}{\partial\chi}+b\frac{\partial(V_{c,max}/A)}{\partial\chi}=0$ (S14)

The parameter *a* is directly proportional to the viscosity of water (*η*), while *b* is generally taken as constant. As the ratio *b*/*a* affects the analytical solution of the optimal *χ*, a composite parameter *β* is used to denote the value of *b*/*a* at 25^o^C. Then, the *b*/*a* at *T*_g_ is quantified as *β*/*η*^*^, where *η*^*^ is the ratio of *η* at *T*_g_ relative to its value at 25^o^C. The parameter *η*^*^ can be calculated using temperature and elevation as in Huber *et al.* (2009) [9], while the composite parameter *β* is often assumed as globally constant, including the model used in Wang *et al.* (2017) [10] and Smith *et al.* (2019) [2]. After a series of derivations, the optimal *C*_i_ can be calculated as Equations (S15-S16):

$\frac{C_{i}}{C_{a}}=\frac{\Gamma^{*}}{C_{a}}+\left( 1-\frac{\Gamma^{*}}{C_{a}} \right)\frac{\xi}{\xi+\sqrt{D_{g}}}$(S15)

$\xi=\sqrt{\beta\frac{K+\Gamma^{*}}{1.6\eta^{*}}}$ (S16)

where *ξ* defines the sensitivity of *C*_i_/*C*_a_ to *D*_g_, and *β* is set as a constant at 146. For more details about the optimal *C*_i_, please refer to Prentice *et al.* (2014) [8] and Wang *et al.* (2017) [10].

Finally, *V*_c,max_ was derived at its *T*_g_, which could further be converted to 25 ℃ and its measurement temperature using Equations (1-3).

**References**

[1] Y.K. Peng, K.J. Bloomfield, L.A. Cernusak, et al., Global climate and nutrient controls of photosynthetic capacity, Commun. Biol. 4 (2021) 462.

[2] N.G. Smith, T.F. Keenan, I.C. Prentice, et al., Global photosynthetic capacity is optimized to the environment, Ecol. Lett. 22 (2019) 506-517.

[3] V. Maire, P. Martre, J. Kattge, et al., The coordination of leaf photosynthesis links C and N fluxes in C3 plant species, PLoS ONE 7 (2012) e38345.

[4] C.J. Bernacchi, E.L. Singsaas, C. Pimentel, et al., Improved temperature response functions for models of Rubisco-limited photosynthesis, Plant Cell Environ. 24 (2001) 253-259.

[5] G.V. Farquhar, S. Wong, An empirical model of stomatal conductance, Funct. Plant Biol. 11 (1983) 191-210.

[6] C.J. Bernacchi, C. Pimentel, S.P. Long, In vivo temperature response functions of parameters required to model RuBP-limited photosynthesis, Plant Cell Environ. 26 (2003) 1419-1430.

[7] N.G. Smith, T.F. Keenan, Mechanisms underlying leaf photosynthetic acclimation to warming and elevated CO_2_ as inferred from least-cost optimality theory, Glob. Change Biol. 26 (2020) 5202-5216.

[8] I.C. Prentice, N. Dong, S.M. Gleason, et al., Balancing the costs of carbon gain and water transport: testing a new theoretical framework for plant functional ecology, Ecol. Lett. 17 (2014) 82-91.

[9] M.L. Huber, R.A. Perkins, A. Laesecke A, et al., New international formulation for the Viscosity of H_2_O, J. Phys. Chem. Ref. Data 38 (2009) 101-125.

[10] H. Wang, I.C. Prentice, T.F. Keenan, et al., Towards a universal model for carbon dioxide uptake by plants, Nat. Plants 3 (2017) 734-741.
